# *In vitro* activity of aztreonam/avibactam and comparators against Enterobacterales isolates from patients with hospital-acquired pneumonia, ventilator-associated pneumonia, and complicated intra-abdominal infections (ATLAS, 2021–2022)

**DOI:** 10.1093/jac/dkag198

**Published:** 2026-06-10

**Authors:** Qiwen Yang, Felipe Francisco Tuon, Sweta Shah, Shweta Kamat, Naglaa Mohamed, Gregory G Stone, Katherine K Perez, Rafael Cantón

**Affiliations:** Department of Clinical Laboratory, State Key Laboratory of Complex Severe and Rare Diseases, Peking Union Medical College Hospital, Chinese Academy of Medical Sciences and Peking Union Medical College, Beijing, China; Laboratory of Emerging Infectious Diseases, Pontifícia Universidade Católica do Paraná, Curitiba, Paraná, Brazil; Department of Microbiology, Kokilaben Dhirubhai Ambani Hospital and Research Institute, Mumbai, India; Pfizer Products India Private Limited, Mumbai, India; Pfizer Inc., NewYork, NY, USA; Pfizer Inc., Groton, CT, USA; Pfizer Inc., NewYork, NY, USA; Servicio de Microbiologia, Hospital Universitario Ramón y Cajal and Instituto Ramón y Cajal de Investigación Sanitaria (IRYCIS), Madrid, Spain; CIBER de Enfermedades Infecciosas (CIBERINFEC), Instituto de Salud Carlos III, Madrid, Spain

## Abstract

**Background:**

Hospital-acquired pneumonia (HAP), ventilator-associated pneumonia (VAP) and complicated intra-abdominal infections (cIAIs) caused by carbapenem-resistant Enterobacterales (CRE) are associated with high mortality. Metallo-β-lactamase (MBL)-producing strains represent therapeutic challenge compromising β-lactams and limiting treatment options. Aztreonam/avibactam is a novel combination designed to overcome MBL-mediated resistance.

**Objectives:**

To evaluate *in vitro* activity of aztreonam/avibactam and comparators against Enterobacterales from patients with HAP, VAP or cIAI collected globally (Africa/Middle East, Asia-Pacific, Europe and Latin America) during 2021–22 ATLAS surveillance program focusing on MBL-positive strains.

**Methods:**

14 564 Enterobacterales isolates were tested by broth microdilution per CLSI guidelines. CRE isolates underwent molecular characterization and MBL-positive isolates underwent additional molecular and susceptibility analysis, including cefiderocol. MBL-positive *Escherichia coli* isolates were screened for PBP3 mutations by whole-genome sequencing. MICs were interpreted using EUCAST breakpoints as appropriate.

**Results:**

Aztreonam/avibactam was 99.5% susceptible (MIC ≤4 mg/L) for all Enterobacterales (MIC_90_ 0.25 mg/L) and maintained high susceptibility against MDR (98.9%), ESBL (99.0%) and CRE (96.4%). Among 577 MBL-positive isolates (88% NDM), aztreonam/avibactam was 95.1% susceptible, compared with cefiderocol (50.3%), colistin (73.8%) and tigecycline (95.0%). Activity was consistent across regions and carbapenemase genotypes, including co-producers of multiple β-lactamases. However, *E. coli* isolates harbouring PBP3 insertions (YRIK/YRIN) and plasmid-mediated AmpC β-lactamases (*n* = 35) were 31.4% susceptible (MIC ≤4 mg/L).

**Conclusions:**

Aztreonam/avibactam demonstrated potent and consistent *in vitro* activity against Enterobacterales causing serious hospital infections, including MBL-producing CRE. These findings underscore aztreonam/avibactam’s therapeutic potential and the need for rapid diagnostics and global surveillance to guide regional stewardship and treatment strategies for MDR infections.

## Introduction

Hospital-acquired pneumonia (HAP), ventilator-associated pneumonia (VAP) and complicated intra-abdominal infections (cIAIs) are among the most serious infections in hospitalized patients, with mortality rates ranging from 20% to 50%, rising to ∼75% when caused by MDR pathogens. These infections lead to poor clinical outcomes, prolonged hospital stays, high costs and reliance on last-resort therapies that may be delayed and cause significant side effects.^1,2^ Carbapenem-resistant Enterobacterales (CRE) are recognized as urgent global AMR threats,^3,4^ driven in part by widespread empiric carbapenem use that has promoted the emergence and dissemination of carbapenemase-producing Enterobacterales.^5,6^ Ambler class A (e.g. KPC) and class D (e.g. OXA-48) serine carbapenemases, together with class B metallo-β-lactamases (MBLs; e.g. NDM, VIM and IMP) account for ∼85% of CRE worldwide.^3,5,6^ CRE infections are associated with high mortality rates (26%–44%),^7^ frequently attributable to delays in effective therapy.^8,9^ Rapid carbapenemase identification and prompt administration of active treatment within robust antimicrobial stewardship programmes are, therefore, critical.^10^

MBL-producing Enterobacterales are of particular concern due to increasing prevalence, molecular diversity and their ability to hydrolyze most frontline β-lactams (including carbapenems) combined with efficient plasmid-mediated dissemination. The highest proportions of MBL-positive Enterobacterales have been reported in Asia-Pacific (APAC) (∼59%) and Africa and Middle East (AfME) (∼49%) regions. Although aztreonam, a monobactam antibiotic, is stable to MBL hydrolysis, it is frequently compromised by co-produced ESBLs and plasmid-mediated AmpC (pAmpC) enzymes reported in ∼81% of isolates.^11^ Additionally, ∼19.5% of MBL-positive CRE globally co-produce multiple β-lactamases,^12^ further limiting treatment options.^3,5,13,14^ Avibactam, a non-β-lactam β-lactamase inhibitor, inhibits class A, C and some D β-lactamases, restoring aztreonam’s activity against MBL-positive CRE, including isolates co-harbouring multiple carbapenemases.^12,14–19^ Aztreonam/avibactam was developed to overcome MBL-mediated resistance.^20,21^ Phase 3 trial data have demonstrated efficacy and safety of aztreonam/avibactam in patients with HAP, VAP or cIAI caused by Gram-negative pathogens.^14,22^ Cefiderocol represents another therapeutic option; however, its activity is compromised in NDM-producing Enterobacterales.^23,24^ Reduced susceptibility and resistance have been reported and linked to impaired siderophore-mediated uptake due to alterations in siderophore receptor/iron-transport pathways required for cefiderocol entry (e.g. CirA disruption), consistent with reduced intracellular access.^25–27^

Aztreonam exerts its antibacterial activity by binding penicillin-binding proteins, particularly penicillin-binding protein 3 (PBP3; also known as FtsI), a key divisome transpeptidase involved in septal peptidoglycan synthesis and bacterial cell division.^28^ Recent reports indicate that PBP3 insertions, particularly YRIK and YRIN tetrapeptide duplications at position 333–337, can reduce aztreonam/avibactam susceptibility in *E. coli* when combined with pAmpC-type β-lactamases.^29,30^

The ATLAS program^11^ has monitored *in vitro* antimicrobial activity worldwide since 2004. In this study, we characterize the *in vitro* activity of aztreonam/avibactam and comparator agents against Enterobacterales isolates from patients with HAP, VAP or cIAI collected in 2021–22 across AfME, APAC, Europe and Latin America (LATAM). MBL-positive isolates underwent additional comparator (including cefiderocol) phenotypic and molecular analysis. Moreover, MBL-positive *E. coli* isolates were screened for PBP3 mutations to further define resistance profiles and therapeutic options.

## Materials and methods

### Bacterial isolates

Predetermined counts of clinical Enterobacterales isolates were collected from non-duplicate patients (adult and paediatric) at participating centres during 2021–22 across AfME, Europe, APAC and LATAM, per ATLAS protocol.^11^ The standardized protocol specifies target numbers of isolates by genus and/or species but does not impose restrictions on infection source or collection unit/wards. Only isolates considered to be causative for infection were included; blood isolates were excluded. Enterobacterales were defined to include *Citrobacter* spp.*, Enterobacter* spp.*, Escherichia* spp.*, Klebsiella* spp.*, Morganella* spp.*, Proteus* spp.*, Providencia* spp. *and Serratia* spp. Isolates were sent to a central reference laboratory [International Health Management Associates (IHMA), Schaumburg, IL, USA IHMA Shanghai site]. Species identification was confirmed using MALDI-TOF MS.

### Antimicrobial susceptibility testing

Minimal inhibitory concentrations (MICs) for aztreonam/avibactam and other antimicrobial agents, including amikacin, aztreonam, cefepime, colistin, meropenem and tigecycline, were determined by broth microdilution, following CLSI guidelines.^31^ Cefiderocol susceptibility testing was not part of the routine panel for ATLAS 2021–22; therefore, testing was completed separately against this MBL-positive Enterobacterales subset of isolates using the EUCAST-approved susceptibility breakpoint of ≤2 mg/L.^32^

For aztreonam/avibactam, MICs were interpreted using the EUCAST-approved susceptibility breakpoint of ≤4 mg/L.^32^ MICs for tigecycline were evaluated based on the susceptibility breakpoint of ≤2 mg/L for Enterobacterales, as established by the FDA.

### Definitions of infections and resistance phenotypes

HAP and VAP were defined as pneumonia ≥48 h after admission or ≥48 h after mechanical ventilation, respectively.^1^ cIAI was defined as infection of gastrointestinal tract that extends beyond, potentially causing peritonitis or abscess formation.^33^ MDR phenotype was defined as resistance to ≥3 antimicrobial classes.^34^ CRE was defined as Enterobacterales resistant to meropenem (MIC >8 mg/L, EUCAST 2025).^32,35^

### Molecular analysis

Meropenem-non-susceptible Enterobacterales isolates (MIC ≥2 mg/L as per CLSI) and a randomly selected subset (>70%) of *E. coli*, *K. pneumoniae*, *K. oxytoca* or *P. mirabilis* isolates with ceftazidime and/or aztreonam MICs ≥1 mg/L were screened for clinically relevant β-lactamase genes by Whole Genome Sequencing (WGS) for China mainland or PCR + Sanger sequencing (Overall).^11^ PCR assays targeted clinically relevant β-lactamase genes, including blaTEM, blaSHV, blaCTX-M groups, blaVEB, blaPER, blaGES, blaOXA-48-like, blaNDM, blaVIM, blaIMP, blaSPM, blaGIM, blaKPC and pAmpC families. MBL-positive *E. coli* isolates were further screened for PBP3 mutations by WGS. Details of the characterization performed are provided in the Supplementary Methods (available as Supplementary data at *JAC* Online).

## Results

A total of 14 564 Enterobacterales isolates from patients with HAP, VAP or cIAI were collected across 60 countries in 2021–22 (AfME, APAC, Europe and LATAM). The collection included mostly *Klebsiella* spp. (*n* = 6711), *Escherichia* spp. (*n* = 3499) and *Enterobacter* spp. (*n* = 1631), and additional details and breakdown are included in Supplementary Material (Table S1). Most isolates (61.4%) were from non-ICU settings (ranging 56.4%–70.0% regionally), with a higher proportion of CRE isolates (56.2%) recovered in ICU settings (Table S2).

### Distribution of Enterobacterales based on phenotypes

**Figure 1.**
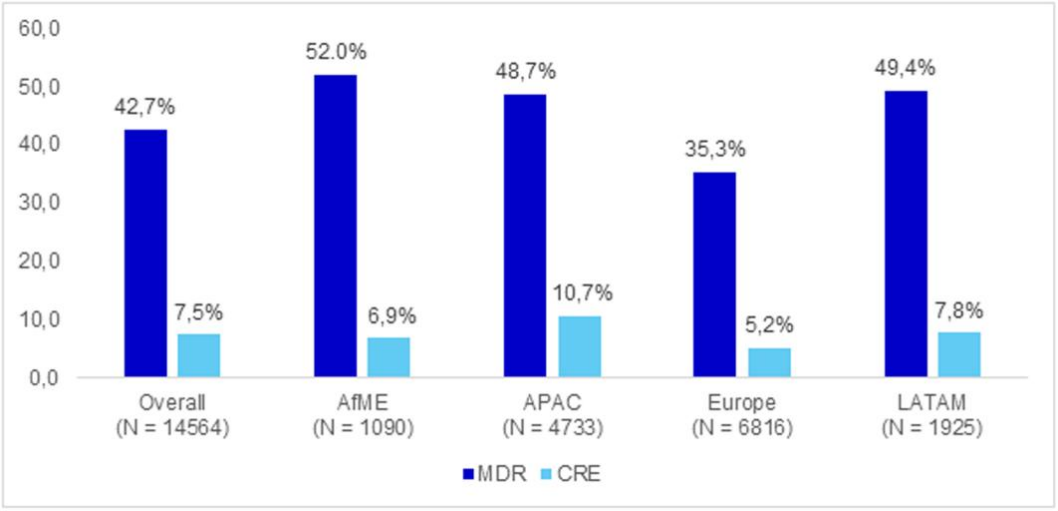
Distribution of MDR (EUCAST) and CRE (EUCAST) phenotype isolates from HAP, VAP and cIAI across regions (2021–22). AfME, Africa and Middle East; APAC, Asia-Pacific; cIAI, complicated intra-abdominal infections; CRE, carbapenem-resistant Enterobacterales; EUCAST, European Committee on Antimicrobial Susceptibility Testing; HAP, hospital-acquired pneumonia; LATAM, Latin America; MDR, multi-drug resistant; VAP, ventilator-acquired pneumonia. Overall captures data from AfME, APAC, Europe and LATAM. Enterobacterales included the following genera: *Citrobacter*, *Enterobacter*, *Escherichia, Klebsiella, Morganella, Providencia, Proteus* and *Serratia*.

**Figure 2.**
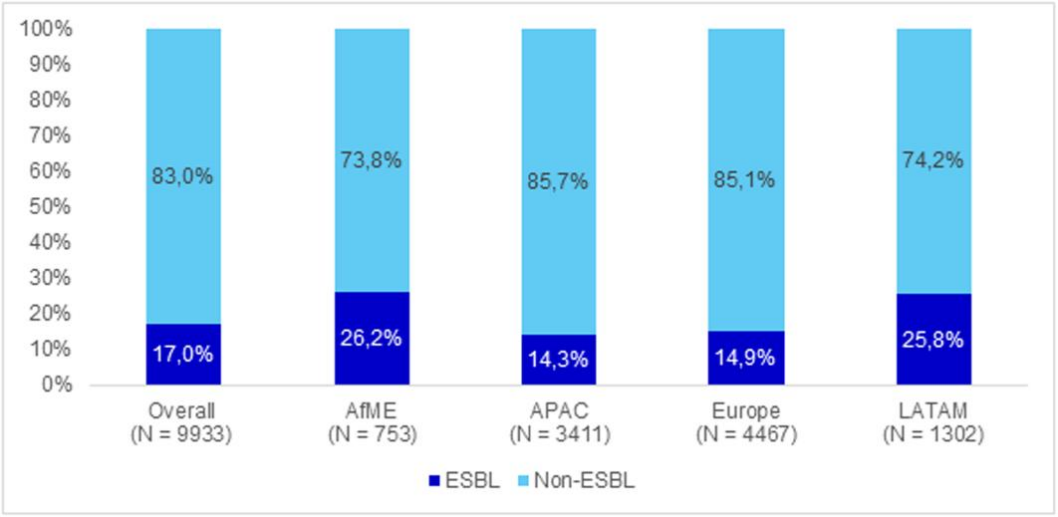
Distribution of ESBL-positive Enterobacterales isolates from HAP, VAP and cIAI across regions (2021–22). AfME, Africa and Middle East; APAC, Asia-Pacific; cIAI, complicated intra-abdominal infections; EUCAST, European Committee on Antimicrobial Susceptibility Testing; ESBL, extended-spectrum β-lactamase; HAP, hospital-acquired pneumonia; LATAM, Latin America; VAP, ventilator-acquired pneumonia. Overall captures data from AfME, APAC, Europe and LATAM. Enterobacterales that qualified for β-lactamase screening: *Escherichia coli, Klebsiella oxytoca, Klebsiella pneumoniae, Klebsiella variicola* and *Proteus mirabilis*.

Overall, 42.7% of the phenotypes met MDR criteria (6217/14 564), with the highest rates observed in AfME (52.0%) and LATAM (49.4%). A total of 1088 CRE isolates were identified (7.5%), with the highest prevalence in APAC (505/4733; 10.7%) followed by LATAM (151/1925; 7.8%), AfME (75/1090; 6.9%) and then Europe (357/6816; 5.2%) (Figures 1 and 2; Tables S2 and S3) ESBL-producing isolates accounted for 17% (1688/9933) and were screened for β-lactamase production with highest proportion found in the AfME region (197/753; 26.2%) (Table S2).

Carbapenemase analysis from the ATLAS database was available for 909/1088 CRE. The regional breakdown of these characterized CRE isolates was: APAC (excluding China mainland) (344/4733; 7.3%), LATAM (142/1925; 7.4%) AfME (73/1090; 6.7%) and then Europe (350/6816; 5.1%). Genotypically, MBL enzymes were the most prevalent carbapenemase (448/909; 49.3%) followed by OXA-48 and OXA-48-like (377/909; 41.5%) and KPC (254/909; 27.9%) (Table 1).

**Table 1.** Distribution of CRE isolates from HAP, VAP and cIAI across regions (2021–22)

	Overall^a^ (*n* = 14 564) [*n* (%)]	AfME (*n* = 1090) [*n* (%)]	APAC (*n* = 4733) [*n* (%)]	Europe (*n* = 6816) [*n* (%)]	LATAM (*n* = 1925) [*n* (%)]
CR-Enterobacterales (*n*)	909	73 (6.7)	344 (7.3)	350 (5.1)	142 (7.4)
MBL	448 (49.3)	61 (83.6)	214 (62.2)	118 (33.7)	55 (38.7)
NDM	408 (44.9)	60 (82.1)	207 (60.2)	89 (25.4)	52 (36.6)
VIM	31 (3.4)	0	0	28 (8.0)	3 (2.1)
IMP	9 (0.1)	1 (1.4)	7 (2.0)	1 (0.3)	0
OXA-48 and OXA-48-like	377 (41.5)	32 (43.8)	220 (63.9)	123 (35.1)	2 (1.4)
KPC	254 (27.9)	0	12 (3.5)	152 (43.4)	90 (63.4)

Enterobacterales included the following genera: *Citrobacter, Enterobacter*, *Escherichia, Klebsiella, Morganella, Providencia, Proteus* and *Serratia.*

AfME, Africa and Middle East; APAC, Asia-Pacific; cIAI, complicated intra-abdominal infections; CRE, carbapenem-resistant Enterobacterales; HAP, hospital-acquired pneumonia; IMP, imipenemase metallo-β-lactamase; KPC, Klebsiella pneumoniae carbapenemase; LATAM, Latin America; NDM, New Delhi metallo-β-lactamase; OXA-48-like, oxacillinase-48-like β-lactamase; VAP, ventilator-acquired pneumonia; VIM, Verona Integron-encoded metallo-β-lactamase.

^a^Overall captures isolates from AfME, APAC excluding China mainland (molecular data were not available within ATLAS for those isolates), Europe and LATAM.

### MBL-positive Enterobacterales

Following the inclusion of isolates with meropenem MIC ≥2 mg/L (as defined by CLSI for CRE) and those collected in China mainland, a total of 577 isolates were classified as MBL-positive. Genotypically, NDM was the predominant enzyme accounting for 88% (508/577) of all MBL-positive isolates. The highest NDM prevalence was observed in AfME (67/68; 98.5%) and LATAM (70/72; 97.2%). Notably, the NDM-5 variant was very common in APAC, representing 57.7% (157/272) of NDM-producing isolates. Other MBL genotypes were less frequent overall: VIM (48/577; 8.3%) was primarily found in Europe (45/145; 31%) and IMP (25/577; 4.3%) with higher prevalence in APAC (23/292; 7.9%) (Table 2).

**Table 2.** Genotype-based distribution of MBL-positive Enterobacterales from HAP, VAP and cIAI across regions (2021–22)

	Overall (*n* = 577) [*n* (%)]	AfME (*n* = 68) [*n* (%)]	APAC (*n* = 292) [*n* (%)]	Europe (*n* = 145) [*n* (%)]	LATAM (*n* = 72) [*n* (%)]
All Enterobacterales (*n* = 14 564)					
NDM^a^	508 (88.0)	67 (98.5)	272 (93.2)	99 (68.3)	70 (97.2)
NDM-1	243 (47.8)	21 (33.3)	86 (31.6)	82 (82.8)	54 (77.1)
NDM-5	221 (43.5)	36 (53.7)	157 (57.7)	16 (16.1)	12 (17.1)
VIM^a^	48 (8.3)	0	0	45 (31.0)	3 (4.2)
IMP^a^	25 (4.3)	1 (1.5)	23 (7.9)	1 (0.7)	0
IMP-4	2 (8.0)	0	2 (8.7)	0	0
IMP-8	19 (76.0)	0	19 (82.6)	0	0
IMP-23	2 (8.0)	0	2 (8.7)	0	0
IMP-Type	2 (8.0)	1 (100)	0	1 (100)	0
Co-carriage with Class A (KPC) or Class D (OXA-48 like)^a^	175 (30.3)	21 (30.9)	109 (37.3)	41 (28.2)	4 (5.6)
OXA-48-like	145 (82.9)	21 (100)	98 (89.9)	25 (61.0)	1 (25.0)
KPC	30 (17.1)	0	11 (10.1)	16 (39.0)	3 (75.0)
Co-carriage with ESBL^b^ (*n* = 450)	353 (78.4)	54 (94.7)	177 (78.3)	85 (72.6)	37 (74)
Co-carriage with pAmpC^b^ (*n* = 450)	72 (16.0)	3 (5.2)	41 (18.1)	21 (17.9)	7 (14)
Co-carriage with PBP3^c^ (*n* = 81)	61 (75.3)	2 (2.5)	55 (67.9)	0	4 (4.9)
YRIK	21 (34.4)	0	21 (38.2)	0	0
YRIN	40 (65.6)	2 (100)	34 (61.8)	0	4 (100)
YRIK/YRIN + pAmpC	35 (57.4)	1 (50.0)	33 (60.0)	0	1 (25.0)

Overall captures data from AfME, APAC (includes China mainland), Europe and LATAM.

AfME, Africa and Middle East; APAC, Asia-Pacific; cIAI, complicated intra-abdominal Infections; CRE, carbapenem-resistant Enterobacterales; ESBL, extended-spectrum β-lactamase; HAP, hospital-acquired Pneumonia; IMP, imipenemase metallo-β-lactamase; KPC, Klebsiella pneumoniae Carbapenemase; LATAM, Latin America; NDM, New Delhi metallo-β-lactamase; OXA-48-like, oxacillinase-48-like β-lactamase; pAmpC, plasmid-mediated ambler class C; β-lactamase; VAP, ventilator-acquired pneumonia; VIM, Verona Integron-encoded metallo-β-lactamase.

^a^Enterobacterales included the following genera: *Citrobacter, Enterobacter*, *Escherichia, Klebsiella, Morganella, Proteus*, *Providencia* and *Serratia*.

^b^Enterobacterales that qualified for β-lactamase screening: *Escherichia coli, Klebsiella oxytoca, Klebsiella pneumoniae, Klebsiella variicola* and *Proteus mirabilis.*

^c^PBP3 mutation analysis (YRIK/YRIN) was performed on *Escherichia coli* isolates.

**Figure 3.**
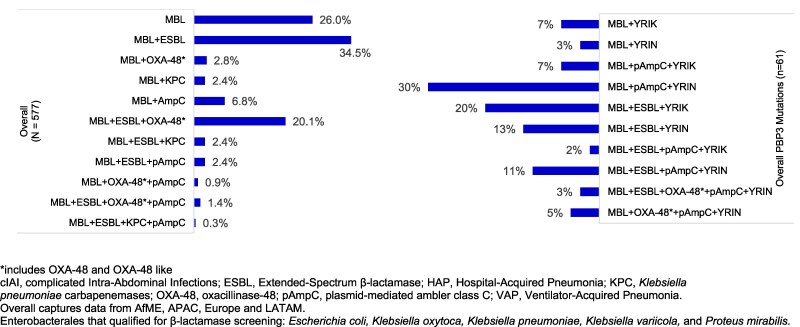
Distribution of various β-lactamases in MBL-positive Enterobacterales isolates from HAP, VAP and cIAI (2021–22). * includes OXA-48 and OXA-48 like cIAI, complicated intra-abdominal infections; ESBL, extended-spectrum β-lactamase; HAP, hospital-acquired pneumonia; KPC, *Klebsiella pneumoniae* carbapenemases; OXA-48, oxacillinase-48; pAmpC, plasmid-mediated ambler class C; VAP, ventilator-acquired pneumonia. Overall captures data from AfME, APAC, Europe and LATAM. Enterobacterales that qualified for β-lactamase screening: *Escherichia coli, Klebsiella oxytoca, Klebsiella pneumoniae, Klebsiella variicola* and *Proteus mirabilis*.

Among the 577 MBL-positive isolates, 30.3% (175/577) co-carried a serine carbapenemase (KPC and/or OXA-48/OXA-48-like enzyme). APAC had the highest co-carriage rate at 37.3% (109/292). Additionally, 450 isolates carried other non-carbapenemase beta-lactamases including ESBLs (353/450; 78.4%) and pAmpC (72/450; 16%) (Table 2, Table S4). The distribution of carbapenemases, including isolates harbouring multiple carbapenemases, is depicted in Figure 3.

### Antimicrobial activity of aztreonam/avibactam and comparators against all Enterobacterales

Aztreonam/avibactam demonstrated potent activity against Enterobacterales from HAP, VAP or cIAI with a 99.5% susceptibility at MIC ≤4 mg/L (MIC_50_ 0.06 mg/L, MIC_90_ 0.25 mg/L), with 12.3% of isolates inhibited at the lowest inhibiting concentration of ≤0.015 mg/L. Comparator agents including ceftazidime/avibactam, meropenem, meropenem-vaborbactam, tigecycline and amikacin also showed high susceptibility (ranging 92%–96%), whereas ceftolozane/tazobactam (82.7%) and colistin (83.2%) showed moderate susceptibility (Table 3). Aztreonam/avibactam’s activity was uniformly high against *Enterobacter cloacae* (99.7% susceptible; MIC_90_ 0.5 mg/L)*, E. coli* (98.8% susceptible; MIC_90_ 0.12 mg/L) and *K. pneumoniae* (99.8% susceptible; MIC_90_ 0.25 mg/L) (Table S5). Regionally, aztreonam/avibactam maintained >99% susceptibility in each region. In APAC, overall susceptibility was 98.9% although 96.7% of *E. coli* isolates were inhibited at ≤4 mg/L. By contrast, some comparators displayed more variability by region. For example, colistin susceptibility was 85.9% in APAC (MIC_90_ 16 mg/L) but lower in LATAM 80.9% (MIC_90_ 16 mg/L) (Table S6A–D).

**Table 3.** MIC distribution and antimicrobial activities of aztreonam/avibactam and comparators against all Enterobacterales from HAP, VAP and cIAI (2021–22)

Antimicrobial agents	MIC (mg/L)		No. of isolates inhibited at respective MIC (mg/L): *n* (Cumulative percentage of isolates at each MIC)															
	%S	%R	MIC_50_	MIC_90_	≤0.015	0.03	0.06	0.12	0.25	0.5	1	2	4	8	16	32	64	128
All Enterobacterales (*n* = 14 564)																		
Amikacin	92.5	7.5	2	8					39 (0.3)	589 (4.3)	4967 (38.4)	5138 (73.7)	2062 (87.8)	675 (92.5)	238 (94.1)	118 (94.9)	48 (95.2)	690 (100)
Aztreonam	70.2	29.8	0.12	128		1824 (12.5)	3352 (35.5)	2991 (56.1)	1037 (63.2)	360 (65.7)	208 (67.1)	219 (68.6)	235 (70.2)	316 (72.4)	496 (75.8)	857 (81.7)	857 (87.5)	1812 (100)
Aztreonam/avibactam	99.5	0.5	0.06	0.25	1793 (12.3)	4006 (39.8)	4594 (71.3)	2080 (85.6)	979 (92.3)	609 (96.5)	251 (98.3)	112 (99.0)	72 (99.5)	31 (99.7)	14 (99.8)	9 (99.9)	7 (99.9)	7 (100)
Cefepime	76.0	24.0	0.12	64				8996 (61.8)	611 (66.0)	411 (68.8)	367 (71.3)	310 (71.4)	379 (76.0)	469 (79.2)	506 (82.7)	555 (86.5)	1959 (100)	
Ceftazidime/avibactam	95.7	4.3	0.12	1		741 (5.1)	2271 (20.7)	5000 (55.0)	3277 (77.5)	1429 (87.3)	729 (92.3)	332 (94.6)	122 (95.4)	33 (95.7)	10 (95.7)	15 (95.8)	15 (95.9)	590 (100)
Ceftolozane/tazobactam	82.7	17.2	0.5	32			15 (0.1)	664 (4.6)	4969 (38.8)	4135 (67.2)	1744 (79.1)	524 (82.7)	364 (85.2)	309 (87.4)	218 (88.8)	1622 (100)		
Ciprofloxacin	70.9	29.0	0.06	8			8288 (56.9)	712 (61.8)	608 (66.0)	724 (71.0)	407 (73.7)	421 (76.6)	312 (78.8)	3091 (100)				
Colistin	83.2	16.7	0.25	16				4688 (32.2)	6525 (77.0)	680 (81.6)	151 (82.7)	80 (83.2)	76 (83.8)	128 (84.6)	2236 (100)			
Meropenem	92.5	7.5	0.06	1			12 171 (83.6)	614 (87.8)	159 (88.9)	106 (89.6)	92 (90.2)	106 (91.0)	109 (91.7)	119 (92.5)	177 (93.7)	911 (100)		
Meropenem/vaborbactam	94.9	5.1	0.06	0.25			12 776 (87.7)	310 (89.8)	118 (90.6)	146 (91.6)	140 (92.6)	115 (93.4)	105 (94.1)	111 (94.9)	123 (95.7)	620 (100)		
Piperacillin/tazobactam	74.5	25.5	4	128						902 (6.2)	1587 (17.1)	4789 (50.0)	2705 (68.5)	864 (74.5)	634 (78.8)	406 (81.6)	391 (84.3)	2286 (100)
Tigecycline^a^	96.2	3.8	0.5	2		3 (0.02)	119 (0.8)	1720 (12.6)	3927 (39.6)	4553 (70.9)	2473 (87.8)	1219 (96.2)	435 (99.2)	88 (99.8)	27 (100)			

Captures data from Africa and Middle East, Asia-Pacific, Europe and LATAM.

Enterobacterales included the following genera: *Citrobacter*, *Enterobacter*, *Escherichia, Klebsiella, Morganella, Proteus*, *Providencia* and *Serratia*.

EUCAST 2025 (v15.0) approved breakpoints have been used in this analysis.

%S data include percentage isolates susceptible at increased exposure.

cIAI, complicated intra-abdominal infections; EUCAST, European Committee on Antimicrobial Susceptibility Testing; HAP, hospital-acquired pneumonia; MIC, minimum inhibitory concentration; MIC_50_, minimum concentration required to inhibit 50% of the organisms; MIC_90_, minimum concentration required to inhibit 90% of the organisms; S, susceptibility; R, resistance; VAP, ventilator-acquired pneumonia.

^a^Data for tigecycline reported here were calculated based on FDA approved breakpoints.

### Antimicrobial activity of aztreonam/avibactam and comparators against various phenotypes

Aztreonam/avibactam showed high activity against MDR, CRE or ESBL-producing phenotypes (Table 4). Among MDR isolates (*n* = 6217), aztreonam/avibactam susceptibility was 98.9% at ≤4 mg/L (MIC_90_ 0.5 mg/L), notably higher than the susceptibilities of most comparators including tigecycline at 93.9%, colistin at 83.0%, amikacin at 82.7% and meropenem at 82.5%. Against 1088 CRE isolates, aztreonam/avibactam remained highly active (96.4% at ≤4 mg/L), compared with 90.2% for tigecycline and 75.9% for colistin. For the 1688 ESBL-producing isolates, aztreonam/avibactam showed 99.0% coverage (Table 4). Across regions, aztreonam/avibactam susceptibility for MDR and ESBL-positive subsets remained ≥97%, while tigecycline and amikacin showed more variability, and colistin displayed wide regional fluctuation. By species, aztreonam/avibactam provided excellent coverage for MDR and ESBL-producing *E. coli* and *K. pneumoniae*. For example, 97%–99% of MDR *E. coli* and *K. pneumoniae* were susceptible to aztreonam/avibactam (MIC_90_ 0.25–0.5 mg/L), whereas colistin susceptibility for MDR *K. pneumoniae* was lower (∼89% susceptible) (Tables S7 and S8).

**Table 4.** Phenotype-based antimicrobial activities of aztreonam/avibactam and comparators against all Enterobacterales from HAP, VAP and cIAI (2021–22)

Phenotype	Antimicrobials	%S (EUCAST)	% R (EUCAST)	MIC (mg/L)		
				MIC_50_	MIC_90_	MIC range
All Enterobacterales (*n* = 14 564)						
MDR (*n* = 6217)	Amikacin	82.7	17.3	2	128	0.25–128
	Aztreonam	30.5	69.5	32	128	0.03–128
	Aztreonam/avibactam	98.9	1.0	0.12	0.5	0.015–128
	Cefepime	43.9	56.0	8	64	0.12–64
	Colistin	83.0	17.0	0.25	16	0.12–16
	Meropenem	82.5	17.5	0.06	32	0.06–32
	Tigecycline*	93.9	6.1	0.5	2	0.03–16
ESBL (*n* = 1688)	Amikacin	66.3	33.6	4	128	0.25–128
	Aztreonam	2.3	97.7	128	128	0.12–128
	Aztreonam/avibactam	99.0	1.0	0.12	0.5	0.015–128
	Cefepime	6.5	93.5	64	64	0.12–64
	Colistin	86.5	13.5	0.25	8	0.12–16
	Meropenem	62.0	38.0	0.5	32	0.06–32
	Tigecycline*	97.2	2.8	0.5	2	0.06–16
CRE (*n* = 1088)	Amikacin	33.3	66.6	64	128	0.5–128
	Aztreonam	7.0	93.0	128	128	0.03–128
	Aztreonam/avibactam	96.4	3.6	0.25	1	0.015–128
	Cefepime	1.0	99.0	64	64	1–64
	Colistin	75.9	24.1	0.25	16	0.12–16
	Meropenem	0	100	32	32	16–32
	Tigecycline*	90.2	9.8	1	2	0.12–16

*Data for tigecycline reported here were calculated based on FDA approved breakpoints. Enterobacterales included the following genera: *Citrobacter, Enterobacter, Escherichia, Klebsiella, Morganella, Proteus, Providencia* and *Serratia*.

cIAI, complicated intra-abdominal infections; CRE, carbapenem-resistant Enterobacterales; EUCAST, European Committee on Antimicrobial Susceptibility Testing; ESBL, extended-spectrum β-lactamase; HAP, hospital-acquired pneumonia; MDR, multi-drug resistant; VAP, ventilator-acquired pneumonia.

### Antimicrobial activity of aztreonam/avibactam and comparators against MBL-positive Enterobacterales

Among 577 MBL-positive isolates, aztreonam/avibactam again demonstrated consistent susceptibility across all genotypes, 95.1% (MIC range: 0.015 to ≥64 mg/L) of isolates at MIC ≤4 mg/L (Table 5). By contrast, cefiderocol inhibited only 50.3% of MBL producers (MIC_90_ 8 mg/L), and colistin 73.8% (MIC_90_ > 8 mg/L) and tigecycline 95.0% (MIC_90_ 2 mg/L).

**Table 5.** MIC distribution and antimicrobial activities of aztreonam/avibactam and comparators against MBL-positive Enterobacterales from HAP, VAP and cIAI (2021–22)

Antimicrobial agent	No. of isolates inhibited at respective MIC (mg/L): *n* (Cumulative percentage of isolates at each MIC)														
	%S	MIC_90_	≤0.015	0.03	0.06	0.12	0.25	0.5	1	2	4	8	16	32	≥64
Overall (*n* = 577)															
Aztreonam	13.9	> 64		8 (1.4)	8 (2.8)	13 (5)	26 (9.5)	12 (11.6)	13 (13.9)	5 (14.7)	12 (16.8)	11 (18.7)	11 (20.6)	26 (25.1)	382 (100)
Aztreonam/ avibactam	95.1	2	9 (1.6)	32 (7.1)	47 (15.3)	76 (28.42)	160 (56.2)	138 (80)	45 (87.9)	19 (91.2)	23 (95.1)	15 (97.7)	7 (99)	5 (99.8)	1 (100)
Cefepime	0.3	>32							2 (0.3)	1 (0.5)	4 (1.2)	6 (2.3)	5 (3.1)	535 (100)	
Colistin	73.8	>8				8 (1.4)	295 (52.5)	111 (71.8)	6 (72.8)	6 (73.8)	4 (74.5)	112 (100)			
Cefiderocol	50.3	8			1 (0.2)	6 (1.2)	6 (2.2)	19 (5.5)	68 (17.3)	190 (50.2)	206 (86.0)	41 (93.0)	16 (95.8)	23 (100)	
Tigecycline^a^	95.0	2				25 (4.3)	158 (31.7)	146 (57.0)	155 (83.9)	64 (95.0)	17 (97.9)	7 (100)			

Captures data from Africa and Middle East, Asia-Pacific, Europe and LATAM.

Enterobacterales included the following genera: *Citrobacter, Enterobacter*, *Escherichia, Klebsiella, Morganella, Proteus*, *Providencia* and *Serratia.*

Enterobacterales that qualified for β-lactamase screening: *Escherichia coli, Klebsiella oxytoca, Klebsiella pneumoniae, Klebsiella variicola* and *Proteus mirabilis.*

EUCAST 2025 (v15.0) approved breakpoints have been used in this analysis.

%S data include percentage isolates susceptible at increased exposure.

cIAI, complicated intra-abdominal infections; EUCAST, European Committee on Antimicrobial Susceptibility Testing; HAP, hospital-acquired pneumonia; MIC, minimum inhibitory concentration; MIC_90_, minimum concentration required to inhibit 90% of the organisms; S, susceptibility; VAP, ventilator-acquired pneumonia.

^a^Data for tigecycline reported here were calculated based on FDA approved breakpoints.

Against NDM, the predominant genotype, aztreonam/avibactam was 94.5% susceptible at ≤4 mg/L (MIC_90_ 2 mg/L) (Table 6). In comparison, the susceptibility of cefiderocol was 47.4% (MIC_90_ 8 mg/L), tigecycline 98.0% (MIC_90_ 2 mg/L) and colistin 74.8% (MIC_90_ > 8 mg/L). Aztreonam/avibactam maintained 100% susceptibility against isolates with VIM and IMP (Table 6 and Table S9). Regionally, aztreonam/avibactam was uniformly active (100%) against NDM-producers in AfME, Europe and LATAM, whereas in APAC, the aztreonam/avibactam susceptibility rate for NDM-producers was slightly lower at 89.7% (APAC MIC_90_ 8 mg/L). Of the 272 NDM-producing isolates from APAC, NDM-5 was most common, comprising approximately 57.7% (157/272), followed by NDM-1 (31.6%). Cefiderocol’s susceptibility against NDM-producers was substantially lower in every region: 64.2% in AfME, 49.3% in APAC, 22.2% in Europe and 60.0% in LATAM (Table S11). Aztreonam/avibactam was fully active against NDM-producing *K. pneumoniae* (100% susceptible; MIC_90_ 0.5 mg/L), but it was less effective against NDM-producing *E. coli* (∼65% susceptible, MIC_90_ 16 mg/L), with particularly reduced susceptibility in APAC, where only ∼55%, MIC_90_ 16 mg/L, of NDM-producing *E. coli* were inhibited (Tables S10 and S11). Many of these aztreonam/avibactam non-susceptible NDM-producing *E. coli* came from a single country (India), where 24/49 tested *E. coli* isolates carried NDM-5 and a pAmpC β-lactamase; aztreonam/avibactam inhibited only 25% of those Indian isolates (Tables S11 and S12).

**Table 6.** Genotype-based antimicrobial activities of aztreonam/avibactam and comparators against MBL-positive Enterobacterales from HAP, VAP and cIAI (2021–22)

Genotypes	*n*	MIC_90_ (mg/L)/% susceptible (%S), EUCAST											
		Aztreonam/avibactam		Aztreonam		Colistin		Cefiderocol		Cefepime		Tigecycline^a^	
		%S	MIC_90_	%S	MIC_90_	%S	MIC_90_	%S	MIC_90_	%S	MIC_90_	%S	MIC_90_
Overall (*n* = 577)													
NDM	508	94.5	2	13.8	>64	74.8	>8	47.4	8	0.0	>32	98.0	2
VIM	48	100.0	2	14.6	>64	72.9	>8	72.9	4	0.0	>32	81.0	4
IMP	25	100.0	2	24.0	>64	60.0	>8	68.0	4	8.0	>32	68.0	>8
Co-carriage													
OXA-48-like	145	98.6	0.5	6.2	>64	73.8	>8	61.4	4	0.0	>32	99.3	2
KPC	30	100.0	1	0.0	>64	60	>8	40.0	16	0.0	>32	86.7	4
ESBL^b^ (*n* = 450)	353	98.9	1	0.6	>64	74.8	>8	50.1	8	0.0	>32	98.6	2
pAmpC^b^ (*n* = 450)	72	66.7	16	2.8	>64	84.7	8	37.5	>32	0.0	>32	94.4	1
YRIK^c^ (*n* = 81)	21	57.1	32	0	>64	100.0	0.5	19.1	4	0.0	>32	100.0	0.5
YRIN^c^ (*n* = 81)	40	52.5	16	2.5	>64	100.0	0.25	20.0	>32	0.0	>32	100.0	0.5
YRIK/YRIN + pAmpC	35	31.4	32	0	>64	100.0	0.25	17.1	>32	0.0	>32	100.0	0.5

Captures data from Africa and Middle East, Asia-Pacific, Europe and LATAM.

Enterobacterales included the following genera: *Citrobacter, Enterobacter, Escherichia, Klebsiella, Morganella, Proteus, Providencia* and *Serratia.*

EUCAST 2025 (v15.0) approved breakpoints have been used in this analysis.

%S data includes percentage isolates susceptible at increased exposure.

cIAI, complicated intra-abdominal infections; EUCAST, European Committee on Antimicrobial Susceptibility Testing; HAP, hospital-acquired pneumonia; MIC, minimum inhibitory concentration; MIC_90_, minimum concentration required to inhibit 90% of the organisms; pAmpC, plasmid-mediated ambler class C; S, susceptibility; VAP, ventilator-acquired pneumonia.

^a^Data for tigecycline reported here were calculated based on FDA approved breakpoints.

^b^Enterobacterales that qualified for β-lactamase screening: *Escherichia coli, Klebsiella oxytoca, Klebsiella pneumoniae, Klebsiella variicola* and *Proteus mirabilis.*

^c^PBP3 mutation analysis was performed on *Escherichia coli* isolates.

Among 81 MBL-positive *E. coli* isolates screened for PBP3 mutations, 61 (75.3%) carried 4-amino-acid insertions at position 333 (YRIN, *n* = 40; YRIK, *n* = 21), while 20 (24.7%) had no known PBP3 insertion. Aztreonam/avibactam susceptibility varied by PBP3 genotype, with lower susceptibility among isolates carrying YRIK or YRIN insertions (57.1%, MIC_90_ 32 mg/L and 52.5%, MIC_90_ 16 mg/L, respectively) compared with isolates without known insertions (100%, MIC_90_ 0.12 mg/L). Aztreonam alone showed very low susceptibility among insertion-bearing isolates (YRIK 0%; YRIN 2.5%) compared with 35% among isolates without known insertions with MIC_90_ of >64 mg/L across groups. Cefiderocol susceptibility was also reduced among isolates with PBP3 insertions (YRIK 19.1%, MIC_90_ 4 mg/L; YRIN 20%, MIC_90_ > 32 mg/L) relative to isolates without known insertions (80%, MIC_90_ 4 mg/L) (Tables 2 and 6). Of 81 *E. coli* isolates, 37 co-harboured pAmpC β-lactamases (predominantly CMY-42 and CMY-145 types) with PBP3 mutations (YRIK, 5/37; YRIN, 30/37; no known insertions, 2/37), with aztreonam/avibactam susceptibility for isolates harbouring PBP3 insertions (YRIK/YRIN) and pAmpC β-lactamases were 31.4% susceptible (MIC_90_ 32 mg/L). (Table 6)

Aztreonam/avibactam also demonstrated 98.6% and 100% susceptibility against isolates co-producing an MBL with OXA-48-like (MBL + OXA-48 and OXA-48 like) or with KPC (MBL + KPC) (MIC_90_ for these subgroups: 0.5 and 1 mg/L, respectively), while tigecycline showed 99.3% susceptibility. Colistin was less effective against MBL + OXA-48 and OXA-48 like isolates (73.8% susceptible; MIC_90_ 8 mg/L), and cefiderocol was much less (61.4% susceptible). In isolates co-harbouring MBL + ESBL enzymes, aztreonam/avibactam inhibited 98.9%, and in those with MBL + pAmpC, aztreonam/avibactam inhibited 66.7% (Table 6). The lower susceptibility against the MBL + pAmpC subset was likely driven by the NDM-5 + YRIK/YRIN + CMY-producing *E. coli* discussed above, e.g. APAC MBL + YRIK/YRIN + pAmpC *E. coli* showed only 27.3% susceptibility to aztreonam/avibactam (Tables S11 and S12). Colistin maintained moderate susceptibility against both MBL + ESBL and MBL + pAmpC isolates (∼75% and 85% susceptible, respectively), whereas cefiderocol was ≤50% active in those subsets (Table 6).

## Discussion

In this global surveillance study, aztreonam/avibactam demonstrated potent and consistent *in vitro* activity against Enterobacterales isolates from patients with HAP, VAP or cIAI in 2021–22 across four major geographic regions, as part of the ATLAS program.^11^ Overall, the results highlight the potent activity of aztreonam/avibactam against MDR, ESBL-producing and CRE isolates, including MBL-positive isolates that represent one of the most challenging resistance profiles in contemporary clinical practice. Overall, aztreonam/avibactam inhibited nearly all Enterobacterales isolates at clinically relevant concentrations, confirming and extending findings from prior ATLAS and SENTRY surveillance analysis.^12,36^

The most clinically significant observation was aztreonam/avibactam’s high activity against MBL-producing Enterobacterales, including isolates co-harbouring serine carbapenemases and additional β-lactamases. In contrast to cefiderocol, which exhibited substantially reduced susceptibility against NDM-producing isolates, aztreonam/avibactam retained activity against the vast majority of MBL-positive CRE. This trend mirrors observations describing the limitations of cefiderocol activity in MBL-producing Enterobacterales, likely related to impaired siderophore-mediated uptake rather than target-based resistance.^26,37,38^ These findings support aztreonam/avibactam as a critical therapeutic option for infections caused by MBL-producing organisms, particularly when alternative agents are unreliable.

Our data also provide updated insight into the global epidemiology of resistance in these serious infections. Nearly half of all isolates met MDR criteria, and CRE accounted for a meaningful proportion of isolates, 7.5% of Enterobacterales overall, with the highest proportion in APAC (10.7%), reflecting the ongoing challenges in managing CRE infections in that region.^35,36,39^ Notably, 61.4% of the isolates were obtained from non-ICU wards, suggesting the presence of resistance beyond critical care settings and supporting the need for hospital and unit-level surveillance.^40,41^ In contrast, CRE isolates were more frequently identified among ICU patients, especially in Europe, consistent with the heightened vulnerability of critically ill populations.^42^ However, these observations reflect the distribution of isolates within the ATLAS dataset and should be interpreted with appropriate caution given the pathogen-based sampling framework.

MBL enzymes, predominantly NDM-producing variants, accounted for ∼45% of carbapenemases among CRE isolates, with particularly high prevalence observed in AfME and APAC. Notably, we observed that NDM-5, a variant associated with enhanced bacterial fitness, is highly prevalent in certain regions, comprising ∼58% of NDM-producing isolates in APAC and ∼54% in AfME, consistent with previous data.^43^ OXA-48/OXA-48-like carbapenemases in Europe and KPC-type carbapenemases were more frequently encountered in LATAM and Europe. These findings reflect geographical clustering of carbapenemases and reinforce the importance of region-specific empiric therapy strategies and continuous surveillance.^12,36,44,45^

Co-resistance among MBL producers was common. Most MBL-positive isolates also carried ESBL (78%) or pAmpC (16%) enzymes, and approximately 30% co-produced an additional carbapenemase. These rates are comparable to other surveillance studies including a more recent increase in co-occurring carbapenemases.^12,36,44,46^ This accumulation of resistance mechanisms underscores the importance of aztreonam/avibactam as a combination capable of addressing this enzymatic complexity. Use of rapid diagnostics to identify resistance mechanisms and antimicrobial stewardship can help optimize treatment outcomes in practice and mitigate resistance development.^15^

Aztreonam/avibactam maintained robust activity against most carbapenemase genotypes and co-resistance profiles examined. However, a subset of NDM-5 producing *E. coli* with PBP3 insertions and pAmpC enzymes exhibited elevated MICs.^47^ These findings are congruent with those of Tellapragada *et al*., reporting 5 NDM-positive *E. coli* harbouring PBP3 amino acid insertions (YRIK/YRIN) and CMY-42 that were resistant to ATM-AVI.^48^ Prior studies indicate that reduced aztreonam/avibactam susceptibility in *E. coli* typically requires the convergence of multiple mechanisms including target alteration (PBP3 modification), enzyme-mediated aztreonam hydrolysis (pAmpC) and possibly reduced permeability rather than a single determinant.^30,49^ Notably, mechanistic investigations of Enterobacterales with elevated aztreonam/avibactam MICs (≥4 mg/L) identified YRIN/YRIK PBP3 insertions in *E. coli* and commonly reported co-carriage of additional β-lactamases such as blaCMY and/or blaCTX-M. Nevertheless, this pattern is not universal: PBP3-modified backgrounds may still exhibit low aztreonam/avibactam MICs, supporting a multi-factorial model in which additional mechanisms are required for decreased susceptibility.^49,50^ Although porin loss was not assessed here, the observed phenotype is consistent with this multi-mechanism model.^12,51^ Experimental data further support that acquired AmpC-type β-lactamases can amplify reduced aztreonam/avibactam susceptibility in PBP3-modified (YRIN/YRIK) *E. coli* backgrounds.^30^ Importantly, such isolates represented a minority of the overall MBL-positive population, and aztreonam/avibactam remained broadly effective even in the presence of extensive β-lactamase co-carriage; additionally, recent work indicates that PBP3 modifications can influence susceptibility to other PBP3-targeting agents, including cefiderocol, supporting the plausibility of cefiderocol shifts observed among insertion-bearing isolates in our analysis.^52^

Several limitations merit consideration. The ATLAS program utilizes predefined pathogen-level isolate targets, which may limit the generalizability of absolute resistance prevalence estimates. Although the protocol does not restrict isolate collection by hospital ward, infection source or timing, the sampling framework may influence the distribution of isolates across clinical settings. As a result, ICU versus non-ICU proportions may not fully reflect underlying epidemiology and should be interpreted as descriptive of the collected dataset rather than population-based estimates. In addition, relatively small sample sizes for certain phenotypes and regions limit the granularity of comparisons. Molecular characterization for all qualifying isolates from China mainland was not readily available and had to be completed as an ancillary project to strengthen the surveillance scope in the APAC region. Integrating data sources from different timelines could introduce minor inconsistencies in the analysis. WGS was not systematically performed (data was added post-hoc), and some resistance determinants, including non-enzymatic mechanisms such as porin loss, may not have been captured. PBP3 screening was limited to *E. coli,* and cefiderocol susceptibility testing was conducted separately on a subset of MBL-positive Enterobacterales rather than as a part of the standard ATLAS panel. Finally, as an *in vitro* surveillance study, no patient-level treatment or outcome data were available. Therefore, *in vitro* activity may not predict clinical outcomes, and clinical correlation remains necessary. Notwithstanding these limitations, the ATLAS program’s (https://atlas-surveillance.com) standardized methodology and centralized laboratory testing provide a robust framework for comparability of antimicrobial susceptibility patterns across regions and over time.

In conclusion, this study confirms the substantial burden of MDR and CRE Enterobacterales in HAP, VAP and cIAI. It also demonstrates that aztreonam/avibactam retains strong *in vitro* activity against these difficult-to-treat pathogens, including nearly all MBL-producing isolates, and generally shows higher activity than other comparator antibiotics. Optimal treatment of CRE infections increasingly depends on identifying the underlying resistance mechanism; however, access to molecular diagnostics is limited in many regions. These findings emphasize the critical need for sustained global surveillance, region-specific antimicrobial stewardship and the integration of novel therapeutic strategies, like aztreonam/avibactam, into clinical practice. Such efforts will be essential to effectively treat patients and curb the escalating threat of resistance in Enterobacterales infections worldwide.

## Supplementary Material

dkag198_Supplementary_Data

## Supplementary data

Tables S1–S12 are available as Supplementary data at *JAC* Online.
