# Supplementary material for: In vitro activity of aztreonam/avibactam and comparators against Enterobacterales isolates from patients with hospital-acquired pneumonia, ventilator-associated pneumonia, and complicated intra-abdominal infections (ATLAS, 2021–2022)
Source: J Antimicrob Chemother. 2026 Jun 10;81(7):dkag198. doi: 10.1093/jac/dkag198 (PMC13250578; doi:10.1093/jac/dkag198)
Supplement: dkag198_Supplementary_Data [file dkag198_supplementary_data.docx]

**SUPPLEMENTARY DATA:**

1. **Supplementary Methods**

The molecular characterization methodology varied by country of collection and reported characteristic (Table 1). In brief, all isolates collected in China were characterized by short-read WGS (Illumina 2x150 bp paired-end), analysis was performed as previously described^1^. Isolates collected in the rest of the world were characterized for the presence of clinically relevant β-lactamase genes using PCR and Sanger sequencing of the full coding sequence as previously described^2^ (Table 2).

Some of the multiplex primers reported by Lob *et al.* to screen for β-lactamase genes were replaced with new primers to enhance detection of less common variants of IMP and PER and to improve multiplexing (CTX-M). New primers were introduced to detect genes encoding GIM.

All variants of β-lactamases, including those encoded by genes detected from WGS and by PCR+Sanger sequencing, were determined using the NCBI Bacterial Antimicrobial Resistance Reference Gene Database (Bioproject 313047). The criterion for calling a β-lactamase variant was a 100% identity match of the full-length deduced amino acid sequence against a sequence in this database.

MBL-positive isolates of *E. coli* collected outside of China were later characterized using long read sequencing (Oxford Nanopore Technologies, Cambridge, United Kingdom) to identify mutations in PBP3. Bacterial isolates were subcultured to tryptic soy agar with 5% sheep’s blood (Remel). Bacterial DNA was isolated using the High Molecular Weight kit (Macherey-Nagel, Düren, Germany). A 1.8x Ampure bead cleanup (Beckman Coulter, Brea, California, USA) was performed on the isolated DNA. The Rapid Barcoding kit (Oxford Nanopore Technologies) was used for library preparation. Sequencing was performed on a Promethion 2 Integrated with R10 flow cells to a coverage depth of 50-100x per barcode. The basecall model was dorado 7.11.2 (super accurate model). Assembly was performed using the Epi2ME bacterial isolates workflow (version 1.4.6). Assemblies were checked for completeness and contamination using the CheckM lineage workflow^3–5^. Pairwise alignment to *E. coli* K12 MG1655 PBP3 (NC_000913.3, b0084) was used to identify insertions in PBP3.

**Table 1. Methodology for molecular characterization**

| Group | Sequencing Method | Analysis reported | Reference |
| --- | --- | --- | --- |
| All isolates collected in China (2021-2022 only)^a^ | Short-read WGS, 2x150 bp paired-end Illumina Hiseq X ten | β-lactamase gene detection  PBP3 mutations | 1 |
| Overall | PCR for clinically-relevant β-lactamase genes. | β-lactamase gene detection | 2 |
| All *E. coli* (ex-China) | Long-read Oxford Nanopore Promethion R10 flow cells, Rapid barcoding kit | PBP3 mutations | This study |

^a^Isolates in China were originally not available for molecular characterization. A subset of meropenem-nonsusceptible isolates from 2021-2022 were later characterized by WGS and the MBL-positive isolates from this group were included in MBL-positive subsets in this manuscript.

**Table 2. Primers used to detect β-lactamase genes**

| **Target Gene** | **Primer name** | **Sequence** | **PCR product size (bp)** | **Reference** |
| --- | --- | --- | --- | --- |
| TEM | TEM-4 | CGTTCATCCATAGTTGCCTGAC | 800 bp | 6 |
| TEM | TEM-3 | CATTTCCGTGTCGCCCTTATTC |  |  |
| SHV | SHV-5 | CCTTTAAAGTAGTGCTCTGC | 119 bp | 2 |
| SHV | SHV-6 | TTCGCTGACCGGCGAGTAGT |  |  |
| VEB | VEB-F | CATTTCCCGATGCAAAGCGT | 648 bp | 6 |
| VEB | VEB-R | CGAAGTTTCTTTGGACTCTG |  |  |
| PER | PERpan-F1 | TAGGCGTTGCAGTGTGGGG | 520 bp | This study |
| PER | PERpan-F2 | TAGGTGTAGCAGTGTGGGG |  |  |
| PER | PERpan-F3 | TAGGTGTTGCCGTATGGGG |  |  |
| PER | PERpan-R1 | GGTTTCGACCATCCACTTCC |  |  |
| PER | PERpan-R2 | GGTTTCAACCATCCATTTCC |  |  |
| GES | GES-F | AGTCGGCTAGACCGGAAAG | 399 bp | 6 |
| GES | GES-R | TTTGTCCGTGCTCAGGAT |  |  |
| OXA-48 Group | OXA-48-F | GCTTGATCGCCCTCGATT | 281 bp | 6 |
| OXA-48 Group | OXA-48-R2 | GATTTGCTSSGTRGCCGAAA |  |  |
| GIM | GIM-F | TCGACACACCTTGGTCTGAA | 477 bp | This study |
| GIM | GIM-R | AACTTCCAACTTTGCCATGC |  |  |
| IMP | IMP2r4 | GGTTTAAYAAARCAACCRCC | 232 bp | This study |
| IMP | IMP2-F | GGAATAGAGTGGCTTAAYTCTC |  |  |
| VIM | VIM-F | GATGGTGTTTGGTCGCATA | 390 bp | 7 |
| VIM | VIM-R3 | CGAATGCGCAGCACCAGGA |  | This study |
| SPM | SPM-F | AAAATCTGGGTACGCAAACG | 271 bp | 7 |
| SPM | SPM-R | ACATTATCCGCTGGAACAGG |  |  |
| NDM | NDM-F | CCGTATGAGTGATTGCGGCG | 779 bp | 8 |
| NDM | NDM-R | GCCCAATATTATGCACCCGG |  |  |
| KPC | KPCy-F | TGTCACTGTATCGCCGTC | 1011 bp | 9 |
| KPC | KPCy-R | CTCAGTGCTCTACAGAAAACC |  |  |
| CMY I/MOX | MOX-MF | GCTGCTCAAGGAGCACAGGAT | 520 bp | 11 |
| CMY I/MOX | MOX-MR | CACATTGACATAGGTGTGGTGC |  |  |
| FOX | FOX-MF | AACATGGGGTATCAGGGAGATG | 190 bp | 11 |
| FOX | FOX-MR | CAAAGCGCGTAACCGGATTGG |  |  |
| ACC | ACC-MF | AACAGCCTCAGCAGCCGGTTA | 346 bp | 11 |
| ACC | ACC-MR | TTCGCCGCAATCATCCCTAGC |  |  |
| CMY II | CIT-MF | TGGCCAGAACTGACAGGCAAA | 462 bp | 11 |
| CMY II | CIT-MR | TTTCTCCTGAACGTGGCTGGC |  |  |
| ACT/MIR | EBC-MF | TCGGTAAAGCCGATGTTGCGG | 302 bp | 11 |
| ACT/MIR | EBC-MR | CTTCCACTGCGGCTGCCAGTT |  |  |
| DHA | DHA-MF | AACTTTCACAGGTGTGCTGGGT | 405 bp | 11 |
| DHA | DHA-MR | CCGTACGCATACTGGCTTTGC |  |  |
| CTX-M-1 | CTX-M1f2 | AAAAATCACTGCGCCAGTTC | 415 bp | 10 |
| CTX-M-1 | CTX-M1r2 | AGCTTATTCATCGCCACGTT |  |  |
| CTX-M-2 | CTX-M2f2 | CGACGCTACCCCTGCTATT | 552 bp | 10 |
| CTX-M-2 | CTX-M2r2 | CCAGCGTCAGATTTTTCAGG |  |  |
| CTX-M-9 | CTX-M9f2 | CAAAGAGAGTGCAACGGATG | 205 bp | 10 |
| CTX-M-9 | CTX-M9r2 | ATTGGAAAGCGTTCATCACC |  |  |
| CTX-M-8 | CTX-M-8A | TCGCGTTAAGCGGATGATGC | 259 bp | 10 |
| CTX-M-8 | CTXM8MR1 | TTGCGTTTCACTTTGCTTG |  | This study |
| CTX-M-25 | CTX-M-25A | GCACGATGACATTCGGG | 249 bp | This study |
| CTX-M-25 | CTXM25MR2 | ACGCTGAGTGTCACCCAGG |  |  |

**References**

1. Estabrook M, Kazmierczak KM, Wise M, *et al*. Molecular characterization of clinical isolates of Enterobacterales with elevated MIC values for aztreonam-avibactam from the INFORM global surveillance study, 2012–2017. *J Glob Antimicrob Resist.* 2021; **24:** 316–320.
2. Lob SH, Kazmierczak KM, Badal RE, *et al*. Trends in Susceptibility of Escherichia coli from Intra-Abdominal Infections to Ertapenem and Comparators in the United States According to Data from the SMART Program, 2009 to 2013. *Antimicrob Agents Chemother* 2015; **59(6):** 3606–10.
3. Parks DH, Imelfort M, Skennerton CT, *et al.* CheckM: assessing the quality of microbial genomes recovered from isolates, single cells, and metagenomes. Genome Research, 2015; **25:** 1043–1055.
4. Matsen FA, Kodner RB, Armbrust EV. pplacer: linear time maximum-likelihood and Bayesian phylogenetic placement of sequences onto a fixed reference tree. *BMC Bioinformatics* 2010; **11:** 538.
5. Hyatt D, Locascio PF, Hauser LJ, Uberbacher EC. Gene and translation initiation site prediction in metagenomic sequences. *Bioinformatics* 2012; **28:** 2223–2230.
6. Dallenne C, Da Costa A, Decre D, *et al.* Development of a set of multiplex PCR assays for the detection of genes encoding important beta-lactamases in Enterobacteriaceae. *J Antimicrob Chemother* 2010; **65:** 490–495.
7. Ellington MJ, Kistler J, Livermore DM, et al. Multiplex PCR for rapid detection of genes encoding acquired metallo-beta-lactamases. *J Antimicrob Chemother* 2007; **59:** 321–322.
8. Lascols C, Hackel M, Marshall SH, et al. Increasing prevalence and dissemination of NDM-1 metallo-β-lactamase in India: data from the SMART study (2009). *J Antimicrob Chemother* 2011; **9:** 1992–1997.
9. Yigit H, Queenan AM, Anderson GJ, *et al.* Novel carbapenem-hydrolyzing betalactamase, KPC-1, from a carbapenem-resistant strain of Klebsiella pneumoniae. *Antimicrob Agents Chemother* 2001; **45:** 1151–1161.
10. Woodford N, Fagan, EJ, Ellington MJ. Multiplex PCR for rapid detection of genes encoding CTX-M extended-spectrum beta-lactamases. *J Antimicrob Chemother* 2006; **57:** 154–155.
11. Perez-Perez FJ and Hanson ND. Detection of plasmid-mediated AmpC beta-lactamase genes in clinical isolates by using multiplex PCR. *J Clinical Microbiol* 2002; **40:** 2153–2162.

1. **Supplementary Tables**

**Supplementary Table 1. Distribution of species of all Enterobacterales from HAP, VAP, and cIAI across regions (2021–2022).**

| **Enterobacterales** | **Overall (N = 14,564) n (%)** | **AfME (N = 1,090) n (%)** | **APAC (N = 4,733) n (%)** | **Europe (N = 6,816) n (%)** | **LATAM (N = 1,925) n (%)** |
| --- | --- | --- | --- | --- | --- |
| ***Citrobacter* spp** | **732 (5.0)** | **59 (5.4)** | **202 (4.3)** | **390 (5.7)** | **81 (4.2)** |
| *C. freundii* | 308 (42.0) | 18 (30.5) | 101 (50.0) | 149 (38.2) | 40 (49.4) |
| *C. koseri* | 293 (40.0) | 34 (57.6) | 76 (37.6) | 161 (41.3) | 22 (27.1) |
| ***Enterobacter* spp** | **1631 (11.2)** | **121 (11.1)** | **485 (10.2)** | **787 (11.5)** | **238 (12.4)** |
| *E. cloacae* | 421 (25.8) | 36 (29.7) | 121 (24.9) | 197 (25.0) | 67 (28.1) |
| ***Escherichia* spp** | **3499 (24.0)** | **263 (24.1)** | **1092 (23.1)** | **1659 (24.3)** | **485 (25.2)** |
| *E. coli* | 3482 (99.5) | 263 (100.0) | 1088 (99.6) | 1648 (99.3) | 483 (99.6) |
| ***Klebsiella* spp** | **6711 (46.1)** | **496 (45.5)** | **2404 (50.8)** | **2979 (43.7)** | **832 (43.2)** |
| *K. oxytoca* | 739 (11.0) | 28 (5.6) | 137 (5.7) | 512 (17.2) | 62 (7.4) |
| *K. pneumoniae* | 5064 (34.8) | 422 (85.1) | 1995 (83.0) | 1970 (66.1) | 677 (81.4) |
| ***Morganella* spp** | **283 (1.9)** | **21 (1.9)** | **67 (1.4)** | **161 (2.4)** | **34 (1.8)** |
| ***Proteus* spp** | **502 (3.4)** | **31 (2.8)** | **134 (2.8)** | **273 (4.0)** | **64 (3.3)** |
| *P. mirabilis* | 350 (69.7) | 24 (77.4) | 98 (73.1) | 178 (65.2) | 50 (78.1) |
| ***Providencia* spp** | **162 (1.1)** | **20 (1.8)** | **39 (0.8)** | **69 (1.0)** | **34 (1.8)** |
| ***Serratia* spp** | **1044 (7.2)** | **79 (7.2)** | **310 (6.5)** | **498 (7.3)** | **157 (8.2)** |
| *S. marcescens* | 806 (77.2) | 62 (78.5) | 222 (71.6) | 395 (79.3) | 127 (80.9) |
| AfME, Africa and Middle East; APAC, Asia-Pacific; cIAI, complicated Intra-Abdominal Infections; HAP, Hospital-Acquired Pneumonia; LATAM, Latin America; VAP, Ventilator-Acquired Pneumonia.  Overall captures data from AfME, APAC, Europe and LATAM | | | | | |

**Supplementary Table 2. Phenotype-based distribution of all Enterobacterales from HAP, VAP, and cIAI across regions (2021-2022).**

| **Enterobacterales** | **Overall (N = 14,564)**  **[n (%)]** | **AfME (N = 1,090) [n (%)]** | **APAC (N = 4,733) [n (%)]** | **Europe (N = 6,816) [n (%)]** | **LATAM (N = 1,925) [n (%)]** |
| --- | --- | --- | --- | --- | --- |
| ICU isolates | 5628 (38.6) | 400 (36.7) | 1421 (30.0) | 2973 (43.6) | 834 (43.3) |
| Non-ICU isolates | 8936 (61.4) | 690 (63.3) | 3312 (70.0) | 3843 (56.4) | 1091 (56.7) |
| **MDR (EUCAST) ^†^** | 6217 (42.7) | 567 (52.0) | 2294 (48.7) | 2404 (35.3) | 952 (49.4) |
| ICU isolates | 2535 (40.8) | 216 (38.1) | 780 (34.0) | 1124 (46.8) | 415 (43.6) |
| Non-ICU isolates | 3682 (59.2) | 351 (61.9) | 1514 (66.0) | 1280 (53.2) | 537 (56.4) |
| **CRE (EUCAST) ^†^** | 1088 (7.5) | 75 (6.9) | 505 (10.7) | 357 (5.2) | 151 (7.8) |
| ICU isolates | 611 (56.2) | 39 (52.0) | 276 (54.7) | 214 (59.9) | 82 (54.3) |
| Non-ICU isolates | 477 (43.8) | 36 (48.0) | 229 (45.3) | 143 (40.9) | 69 (45.7) |
| **ESBL^‡^ (N = 9933)** | 1688 (17.0) | 197 (26.2) | 488 (14.3) | 667 (14.9) | 336 (25.8) |
| ICU isolates | 827 (49.0) | 80 (40.6) | 255 (52.2) | 341 (51.1) | 151 (44.9) |
| Non-ICU isolates | 861 (51.0) | 117 (59.4) | 233 (47.8) | 326 (48.9) | 185 (55.1) |
| AfME, Africa and Middle East; APAC, Asia-Pacific; cIAI, complicated Intra-Abdominal Infections; CRE, Carbapenem-Resistant Enterobacterales; ESBL, Extended-Spectrum β-lactamase; HAP, Hospital-Acquired Pneumonia; LATAM, Latin America; MDR, Multi-Drug Resistant; VAP, Ventilator-Acquired Pneumonia.  Overall captures data from AfME, APAC, Europe and LATAM.  **^†^**Enterobacterales included the following genera: *Citrobacter,* *Enterobacter,* *Escherichia, Klebsiella, Morganella, Proteus*, *Providencia,* and*Serratia*.  ^‡^Enterobacterales that qualified for β-lactamase screening: *Escherichia coli, Klebsiella oxytoca, Klebsiella pneumoniae, Klebsiella variicola,* and *Proteus mirabilis.* | | | | | |

**Supplementary Table 3. Phenotype-based distribution of species of all Enterobacterales from HAP, VAP, and cIAI across regions (2021–2022).**

| **Enterobacterales** | **Overall** **(N = 14,564)**  **n (%)** | **AfME** **(N = 1090)**  **n (%)** | **APAC** **(N = 4733)**  **n (%)** | **Europe** **(N = 6816)**  **n (%)** | **LATAM** **(N = 1925)**  **n (%)** |
| --- | --- | --- | --- | --- | --- |
| ***Citrobacter freundii*** | **308 (2.1)** | **18 (1.6)** | **101 (2.1)** | **149 (2.2)** | **40 (2.1)** |
| MDR | 128 (41.5) | 4 (22.2) | 51 (50.5) | 53 (35.6) | 20 (50.0) |
| CRE | 17 (5.5) | 1 (5.5) | 9 (8.9) | 3 (2.0) | 4 (10.0) |
| ***Citrobacter koseri*** | **293 (2.0)** | **34 (3.1)** | **76 (1.6)** | **161 (2.3)** | **22 (1.1)** |
| MDR | 15 (5.1) | 1 (2.9) | 9 (11.8) | 4 (2.5) | 1 (4.5) |
| ***Escherichia coli*** | **3482 (23.9)** | **263 (24.1)** | **1088 (23.0)** | **1648 (24.2)** | **483 (25.1)** |
| MDR | 1729 (49.6) | 152 (57.8) | 714 (65.6) | 581 (35.2) | 282 (58.4) |
| CRE | 75 (2.1) | 1 (0.4) | 59 (5.4) | 2 (0.1) | 13 (2.7) |
| ESBL | 401 (11.5) | 45 (17.1) | 113 (10.4) | 135 (8.2) | 108 (22.4) |
| ***Klebsiella pneumoniae*** | **5064 (34.8)** | **422 (38.7)** | **1995 (42.1)** | **1970 (28.9)** | **677 (35.2)** |
| MDR | 2545 (50.2) | 279 (66.1) | 909 (45.6) | 962 (48.8) | 395 (58.3) |
| CRE | 893 (17.6) | 67 (15.9) | 399 (20.0) | 325 (16.5) | 102 (15.1) |
| ESBL | 1251 (24.7) | 150 (35.5) | 367 (18.4) | 510 (25.9) | 224 (33.1) |
| ***Klebsiella oxytoca*** | **739 (5.0)** | **28 (2.6)** | **137 (2.9)** | **512 (7.5)** | **62 (3.2)** |
| MDR | 104 (14.1) | 2 (7.1) | 25 (18.2) | 68 (13.3) | 9 (14.5) |
| CRE | 5 (0.7) | 0 | 2 (1.4) | 2 (0.4) | 1 (1.6) |
| ESBL | 12 (1.6) | 0 | 1 (0.7) | 9 (1.7) | 2 (3.2) |
| ***Morganella spp*** | **283 (1.9)** | **21 (1.9)** | **67 (1.4)** | **161 (2.3)** | **34 (1.8)** |
| MDR | 82 (29.0) | 6 (28.6) | 27 (40.3) | 36 (22.4) | 13 (38.2) |
| CRE | 1 (0.3) | 0 | 0 | 0 | 1 (2.9) |
| ***Proteus mirabilis*** | **350 (2.4)** | **24 (2.2)** | **98 (2.1)** | **178 (2.6)** | **50 (2.6)** |
| MDR | 137 (39.1) | 11 (45.8) | 55 (56.1) | 57 (32.0) | 14 (28.0) |
| CRE | 1 (0.3) | 0 | 0 | 1 (0.5) | 0 |
| ESBL | 16 (4.6) | 2 (8.3) | 6 (6.1) | 7 (3.9) | 1 (2.0) |
| ***Providencia spp*** | **162 (1.1)** | **20 (1.8)** | **39 (0.8)** | **69 (1.0)** | **34 (1.8)** |
| MDR | 74 (45.7) | 12 (60.0) | 20 (51.3) | 25 (36.2) | 17 (50.0) |
| CRE | 13 (8.0) | 2 (10.0) | 2 (5.1) | 5 (7.2) | 4 (11.8) |
| ***Serratia marcescens*** | **806 (5.5)** | **62 (5.7)** | **222 (4.7)** | **395 (5.8)** | **127 (6.6)** |
| MDR | 94 (11.7) | 7 (11.3) | 42 (18.9) | 20 (5.0) | 25 (19.7) |
| CRE | 19 (2.3) | 0 | 8 (3.6) | 1 (0.2) | 10 (7.9) |
| AfME, Africa and Middle East; APAC, Asia-Pacific; cIAI, complicated Intra-Abdominal Infections; CRE, Carbapenem-Resistant Enterpbacterales; ESBL, Extended-Spectrum β-lactamase; HAP, Hospital-Acquired Pneumonia; LATAM, Latin America; MDR, Multi-Drug Resistant; Spp, All species; VAP, Ventilator-Acquired Pneumonia.  *Overall captures data from AfME, APAC, Europe and LATAM.  EUCAST definitions have been used for MDR and CRE. | | | | | |

**Supplementary Table 4: Genotype-based distribution of MBL-positive *E.coli* and *K. pneumoniae* isolates from HAP, VAP, and cIAI across regions (2021-2022).**

|  | **Overall (N = 577) [n (%)]** | **AfME (N = 68) [n (%)]** | **APAC (N = 292) [n (%)]** | **Europe (N = 145) [n (%)]** | **LATAM (N = 72) [n (%)]** |
| --- | --- | --- | --- | --- | --- |
| ***Escherichia coli*** | **81** | **2** | **64** | **0** | **15** |
| NDM | 80 (98.8) | 2 (100.0) | 63 (98.4) | 0 | 15 (100.0) |
| VIM | 0 | 0 | 0 | 0 | 0 |
| IMP | 1 (1.2) | 0 | 1 (1.6) | 0 | 0 |
| Co-carriage with Class A (OXA-48-like) | 4 (4.9) | 1 (50.0) | 2 (3.1) | 0 | 1 (6.7) |
| Co-carriage with Class A (KPC) | 0 | 0 | 0 | 0 | 0 |
| Co-carriage with ESBL | 41 (50.6) | 1 (50.0) | 32 (50.0) | 0 | 8 (53.3) |
| Co-carriage with pAmpC | 37 (45.7) | 1 (50.0) | 34 (53.1) | 0 | 2 (13.3) |
| ***Klebsiella pneumoniae*** | **356** | **55** | **160** | **110** | **31** |
| NDM | 324 (91.0) | 55 (100.0) | 157 (98.1) | 82 (74.5) | 30 (96.8) |
| VIM | 29 (8.1) | 0 | 0 | 28 (25.4) | 1 (3.2) |
| IMP | 3 (0.8) | 0 | 3 (1.9) | 0 | 0 |
| Co-carriage with Class A (OXA-48-like) | 133 (37.3) | 20 (36.3) | 91 (56.9) | 22 (20.0) | 0 |
| Co-carriage with Class A (KPC) | 28 (7.8) | 0 | 11 (6.9) | 15 (13.6) | 2 (6.4) |
| Co-carriage with ESBL | 306 (86.0) | 53 (96.4) | 144 (90.0) | 81 (73.6) | 28 (90.3) |
| Co-carriage with pAmpC | 30 (8.4) | 2 (3.6) | 6 (3.7) | 17 (15.5) | 5 (16.1) |
| AfME, Africa and Middle East; APAC, Asia-Pacific; cIAI, complicated Intra-Abdominal Infections; ESBL, Extended-Spectrum β-lactamase; HAP, Hospital-Acquired Pneumonia; IMP, Imipenemase Metallo-β-lactamase; KPC, Klebsiella pneumoniae Carbapenemase; LATAM, Latin America; NDM, New Delhi Metallo-β-lactamase; MBL, Metallo-β-lactamase; OXA-48-like, Oxacillinase-48-like β-lactamase; pAmpC, plasmid-mediated ambler class C β-lactamase; VAP, Ventilator-Acquired Pneumonia; VIM, Verona Integron-encoded.  *Overall captures data from AfME, APAC, Europe and LATAM. | | | | | |

**Supplementary Table 5: MIC distribution and antimicrobial activities of aztreonam/avibactam and comparators for *E. cloacae,* *E.coli* and *K.pneumoniae* isolates from HAP, VAP and cIAI (2021-2022).**

| **Antimicrobials** | **Susceptibility** | | **MIC (mg/L)** | | **No. of isolates inhibited at respective MIC (mg/L); n (Cumulative percentage of isolates at each MIC)** | | | | | | | | | | |
| --- | --- | --- | --- | --- | --- | --- | --- | --- | --- | --- | --- | --- | --- | --- | --- |
|  | **%S** | **%R** | **MIC_50_** | **MIC_90_** | **≤0.06** | **0.12** | **0.25** | **0.5** | **1** | **2** | **4** | **8** | **16** | **32** | **≥64** |
| ***Enterobacter cloacae (N =421)*** | | | | | | | | | | | | | | | |
| Aztreonam/  avibactam | 99.7 | 0.2 | 0.06 | 0.5 | 235 (55.8) | 73 (73.1) | 39 (82.4) | 48 (93.8) | 18 (98.1) | 6  (99.5) | 1  (99.7) | 0 (99.7) | 1  (100) | - | - |
| Aztreonam | 67.2 | 32.8 | 0.12 | 64 | 108 (25.6) | 112 (52.2) | 34 (60.3) | 10 (62.7) | 5 (63.9) | 8 (65.8) | 6 (67.2) | 9 (69.3) | 18 (73.6) | 36 (82.2) | 75 (100) |
| Cefepime | 84.1 | 15.2 | 0.12 | 32 | - | 258 (61.3) | 18 (65.5) | 18 (69.8) | 20 (74.6) | 23 (80.0) | 17 (84.1) | 12 (86.9) | 10 (89.3) | 15 (92.9) | 30 (100) |
| Colistin | 81.5 | 18.5 | 0.25 | 16 | - | 101 (24.0) | 221 (76.5) | 14 (79.8) | 5 (81) | 2 (81.5) | 5 (82.6) | 8 (84.5) | 65 (100) | - | - |
| Tigecycline* | 97.2 | 2.8 | 0.5 | 1 | - | 6 (1.4) | 75 (19.2) | 263 (81.7) | 51 (93.8) | 14 (97.1) | 11 (99.7) | 1 (100) | - | - | - |
| Meropenem | 96.9 | 3.1 | 0.06 | 0.12 | 349 (82.9) | 37 (91.7) | 6 (93.1) | 4 (94.0) | 2 (94.5) | 2 (95.0) | 4 (96.0) | 4 (96.9) | 4 (97.9) | 9 (100) | - |
| Amikacin | 97.4 | 2.6 | 1 | 4 | - | - | - | 9 (2.1) | 206 (51.1) | 160 (89.1) | 26 (95.2) | 9 (97.4) | 5 (98.6) | 1 (98.8) | 5 (100) |
| ***Escherichia coli (N=3482)*** | | | | | | | | | | | | | | | |
| Aztreonam/  avibactam | 98.8 | 1.2 | 0.03 | 0.12 | 3053 (87.7) | 228 (94.2) | 40 (95.4) | 32 (96.3) | 17 (96.8) | 35 (97.8) | 34 (98.8) | 29 (99.6) | 9 (99.9) | 2 (99.9) | 3 (100) |
| Aztreonam | 70.1 | 29.9 | 0.12 | 64 | 1059 (30.4) | 967 (58.2) | 166 (62.9) | 47 (64.3) | 47 (65.6) | 56 (67.2) | 99 (70.1) | 173 (75.1) | 175 (80.1) | 264 (87.7) | 429 (100) |
| Cefepime | 75.6 | 24.4 | 0.12 | 64 | - | 2158  (62.0) | 103  (64.9) | 58  (66.6) | 73  (68.7) | 99 (71.5) | 141 (75.6) | 142 (79.7) | 126 (83.3) | 147 (87.5) | 435 (100) |
| Colistin | 99.4 | 0.6 | 0.12 | 0.25 | - | 1761 (50.6) | 1524 (94.3) | 151 (98.7) | 15 (99.1) | 11 (99.4) | 15 (99.9) | 1 (99.9) | 4 (100) | - | - |
| Tigecycline* | 99.7 | 0.3 | 0.25 | 0.5 | 112 (3.2) | 1461 (45.2) | 1326 (83.2) | 428 (95.5) | 123 (99.1) | 21 (99.7) | 9 (99.9) | 2 (100) | - | - | - |
| Meropenem | 97.8 | 2.1 | 0.06 | 0.06 | 3287 (94.4) | 47 (95.7) | 18 (96.3) | 12 (96.6) | 13 (97.0) | 7 (97.2) | 15 (97.6) | 8 (97.8) | 14 (98.2) | 61 (100) | - |
| Amikacin | 96.2 | 3.7 | 2 | 8 | - | - | 6 (0.2) | 10 (0.4) | 334 (10.0) | 1781 (61.2) | 880 (86.5) | 340 (96.2) | 76 (98.4) | 17 (98.9) | 38 (100) |
| ***Klebsiella pneumoniae (N = 5064)*** | | | | | | | | | | | | | | | |
| Aztreonam/  avibactam | 99.8 | 0.2 | 0.06 | 0.25 | 3182 (62.8) | 841 (79.4) | 565 (90.6) | 314 (96.8) | 104 (99.0) | 33 (99.5) | 14 (99.8) | 2 (99.8) | 1 (99.8) | 3 (99.9) | 5 (100) |
| Aztreonam | 56.4 | 43.6 | 0.25 | 128 | 1895 (37.4) | 534 (48.0) | 197 (51.9) | 80 (53.4) | 52 (54.4) | 48 (55.4) | 49 (56.4) | 61 (57.6) | 137 (60.3) | 306 (66.3) | 1705 (100) |
| Cefepime | 58.1 | 41.9 | 0.25 | 64 | - | 2464 (48.6) | 151 (51.6) | 95 (53.5) | 94 (55.4) | 55 (56.5) | 84 (58.1) | 230 (62.7) | 289 (68.3) | 309 (74.5) | 1293 (100) |
| Colistin | 94.3 | 5.6 | 0.25 | 0.5 | - | 1314 (25.9) | 3053 (86.2) | 296 (92.1) | 76 (93.6) | 39 (94.3) | 38 (95.1) | 87 (96.8) | 161 (100) | - | - |
| Tigecycline | 95.3 | 4.7 | 0.5 | 2 | 1 (0.02) | 51 (1.0) | 1079 (22.3) | 2040 (62.6) | 1151 (85.3) | 503 (95.3) | 174 (98.7) | 46 (99.6) | 19 (100) | - | - |
| Meropenem | 82.4 | 17.6 | 0.06 | 32 | 3742 (73.9) | 93 (75.7) | 45 (76.6) | 39 (77.4) | 56 (78.5) | 74 (80.0) | 52 (81.0) | 70 (82.4) | 118 (84.7) | 775 (100) | - |
| Amikacin | 84.6 | 15.4 | 1 | 128 | - | - | 18 (0.3) | 374 (7.7) | 2321 (53.6) | 943 (72.2) | 477 (81.6) | 151 (84.6) | 109 (86.7) | 74 (88.2) | 597 (100) |
| cIAI, complicated Intra-Abdominal Infections; EUCAST, European Committee on Antimicrobial Susceptibility Testing; HAP, Hospital-Acquired Pneumonia; MIC, Minimum Inhibitory Concentration; MIC_50_, minimum concentration required to inhibit 50% of the organisms; MIC_90_, minimum concentration required to inhibit 90% of the organisms; S, Susceptibility; R, Resistance; VAP, Ventilator-Acquired Pneumonia  Captures data from Africa and Middle East, Asia-Pacific, Europe and LATAM.  EUCAST 2025 (v15.0) approved breakpoints have been used in this analysis.  %S data includes percentage isolates susceptible at increased exposure. *Data for tigecycline reported here were calculated based on FDA approved breakpoints. | | | | | | | | | | | | | | | |

**Supplementary Table 6: Antimicrobial activities of aztreonam/avibactam and comparators for *E. cloacae,* *E. coli* and *K. pneumoniae* isolates from HAP, VAP and cIAI across regions (2021-2022).**

1. **Africa/Middle East Region**

| **Antimicrobial agent** | **Susceptibility** | | **MIC (mg/L)** | | |
| --- | --- | --- | --- | --- | --- |
|  | **%S** | **%R** | **MIC_50_** | **MIC_90_** | **MIC range** |
| **All Enterobacterales (N = 1090)** | | | | | |
| Aztreonam/avibactam | 99.6 | 0.4 | 0.06 | 0.25 | 0.015–128 |
| Aztreonam | 62.3 | 37.7 | 0.25 | 128 | 0.03–128 |
| Cefepime | 68.0 | 32.0 | 0.12 | 64 | 0.12–64 |
| Colistin | 83.4 | 16.6 | 0.25 | 16 | 0.12–16 |
| Tigecycline* | 94.6 | 5.4 | 0.5 | 2 | 0.03–8 |
| Meropenem | 93.1 | 6.9 | 0.06 | 1 | 0.06–32 |
| Amikacin | 91.6 | 8.3 | 2 | 8 | 0.25–128 |
| ***Enterobacter cloacae*** | | | | | |
| Aztreonam/avibactam | 100 | 0 | 0.06 | 0.5 | 0.015–1 |
| Aztreonam | 63.9 | 36.1 | 0.12 | 32 | 0.06–128 |
| Cefepime | 83.3 | 16.7 | 0.12 | 32 | 0.12–64 |
| Colistin | 66.7 | 33.3 | 0.25 | 16 | 0.12–16 |
| Tigecycline* | 94.4 | 5.6 | 0.5 | 2 | 0.25-4 |
| Meropenem | 100 | 0 | 0.06 | 0.12 | 0.06–2 |
| Amikacin | 94.4 | 5.5 | 1 | 4 | 0.5–16 |
| ***Escherichia coli*** | | | | | |
| Aztreonam/avibactam | 99.6 | 0.4 | 0.03 | 0.06 | 0.015–8 |
| Aztreonam | 64.3 | 35.7 | 0.12 | 64 | 0.03–128 |
| Cefepime | 70.0 | 30.0 | 0.12 | 64 | 0.12–64 |
| Colistin | 99.6 | 0.4 | 0.25 | 0.25 | 0.12–4 |
| Tigecycline* | 99.6 | 0.4 | 0.25 | 0.5 | 0.03–8 |
| Meropenem | 99.6 | 0.4 | 0.06 | 0.06 | 0.06–32 |
| Amikacin | 95.4 | 4.5 | 2 | 8 | 0.25–64 |
| ***Klebsiella pneumoniae*** | | | | | |
| Aztreonam/avibactam | 99.7 | 0.2 | 0.06 | 0.25 | 0.015–128 |
| Aztreonam | 41.2 | 58.8 | 32 | 128 | 0.03–128 |
| Cefepime | 46.4 | 53.5 | 8 | 64 | 0.12–64 |
| Colistin | 95.0 | 5.0 | 0.25 | 0.5 | 0.12–16 |
| Tigecycline* | 97.6 | 2.4 | 0.5 | 2 | 0.12-8 |
| Meropenem | 84.1 | 15.9 | 0.06 | 32 | 0.06–32 |
| Amikacin | 85.3 | 14.7 | 2 | 16 | 0.25–128 |
| ***Proteus mirabilis*** | | | | | |
| Aztreonam/avibactam | 100 | 0 | 0.01 | 0.01 | 0.015–0.25 |
| Aztreonam | 95.8 | 4.2 | 0.03 | 1 | 0.03–32 |
| Cefepime | 83.3 | 16.7 | 0.12 | 32 | 0.12–32 |
| Colistin | 0 | 100 | 16 | 16 | 16 |
| Tigecycline* | 70.8 | 29.2 | 2 | 4 | 0.5–4 |
| Meropenem | 100 | 0 | 0.06 | 0.12 | 0.06–0.12 |
| Amikacin | 91.7 | 8.3 | 4 | 8 | 1–16 |
| ***Serratia marcescens*** | | | | | |
| Aztreonam/avibactam | 100 | 0 | 0.06 | 0.12 | 0.015–0.25 |
| Aztreonam | 91.9 | 8.0 | 0.12 | 0.5 | 0.03–128 |
| Cefepime | 93.5 | 6.4 | 0.12 | 1 | 0.12–64 |
| Colistin | 9.7 | 90.3 | 16 | 16 | 0.5–16 |
| Tigecycline* | 96.8 | 3.2 | 1 | 2 | 0.25–8 |
| Meropenem | 100 | 0 | 0.06 | 0.12 | 0.06–1 |
| Amikacin | 96.8 | 3.2 | 2 | 4 | 0.5–128 |

1. **Asia-Pacific Region**

| **Antimicrobial agent** | **Susceptibility** | | **MIC (mg/L)** | | |
| --- | --- | --- | --- | --- | --- |
|  | **%S** | **%R** | **MIC_50_** | **MIC_90_** | **MIC range** |
| **All Enterobacterales (N =4733)** | | | | | |
| Aztreonam/avibactam | 98.9 | 1.1 | 0.06 | 0.5 | 0.015–128 |
| Aztreonam | 66.0 | 33.9 | 0.12 | 128 | 0.03–128 |
| Cefepime | 71.9 | 28.1 | 0.12 | 64 | 0.12–64 |
| Colistin | 85.9 | 14.1 | 0.25 | 16 | 0.12-16 |
| Tigecycline* | 94.0 | 6 | 0.5 | 2 | 0.06–16 |
| Meropenem | 89.3 | 10.7 | 0.06 | 16 | 0.06–32 |
| Amikacin | 89.7 | 10.3 | 2 | 16 | 0.25–128 |
| ***Enterobacter cloacae*** | | | | | |
| Aztreonam/avibactam | 99.2 | 0.8 | 0.06 | 0.5 | 0.015–16 |
| Aztreonam | 66.9 | 33.0 | 0.25 | 64 | 0.03–128 |
| Cefepime | 80.2 | 19.8 | 0.12 | 32 | 0.12–64 |
| Colistin | 71.1 | 28.9 | 0.25 | 16 | 0.12–16 |
| Tigecycline* | 97.5 | 2.5 | 0.5 | 1 | 0.12–8 |
| Meropenem | 94.2 | 5.8 | 0.06 | 0.12 | 0.06–32 |
| Amikacin | 96.7 | 3.3 | 2 | 4 | 0.5–128 |
| ***Escherichia coli*** | | | | | |
| Aztreonam/avibactam | 96.7 | 3.3 | 0.03 | 0.25 | 0.015–64 |
| Aztreonam | 56.0 | 44.0 | 2 | 128 | 0.03–128 |
| Cefepime | 64.8 | 35.2 | 0.5 | 64 | 0.12–64 |
| Colistin | 99.4 | 0.6 | 0.12 | 0.5 | 0.12–4 |
| Tigecycline* | 90.7 | 9.3 | 0.25 | 0.5 | 0.06–8 |
| Meropenem | 94.6 | 5.4 | 0.06 | 0.12 | 0.06–32 |
| Amikacin | 94.7 | 5.3 | 2 | 8 | 0.5–128 |
| ***Klebsiella pneumoniae*** | | | | | |
| Aztreonam/avibactam | 99.6 | 0.4 | 0.06 | 0.5 | 0.015–128 |
| Aztreonam | 60.8 | 39.2 | 0.12 | 128 | 0.03–128 |
| Cefepime | 62.3 | 37.7 | 0.12 | 64 | 0.12–64 |
| Colistin | 98.0 | 1.9 | 0.25 | 0.5 | 0.12–16 |
| Tigecycline* | 92.9 | 7.1 | 0.5 | 2 | 0.06–16 |
| Meropenem | 80.0 | 20.0 | 0.06 | 32 | 0.06–32 |
| Amikacin | 81.8 | 18.2 | 1 | 128 | 0.25–128 |
| ***Proteus mirabilis*** | | | | | |
| Aztreonam/avibactam | 98.0 | 2.0 | 0.015 | 0.06 | 0.015–32 |
| Aztreonam | 91.8 | 8.2 | 0.03 | 2 | 0.03–128 |
| Cefepime | 84.7 | 15.3 | 0.12 | 8 | 0.12–64 |
| Colistin | 0 | 100 | 16 | 16 | 8–16 |
| Tigecycline | 42.9 | 57.1 | 4 | 8 | 0.5–8 |
| Meropenem | 100 | 0 | 0.06 | 0.12 | 0.06–1 |
| Amikacin | 80.6 | 19.4 | 4 | 128 | 2–128 |
| ***Serratia marcescens*** | | | | | |
| Aztreonam/avibactam | 99.5 | 0.5 | 0.12 | 0.25 | 0.015–32 |
| Aztreonam | 84.7 | 15.3 | 0.12 | 64 | 0.03–128 |
| Cefepime | 86.0 | 14.0 | 0.12 | 16 | 0.12–64 |
| Colistin | 5.9 | 94.1 | 16 | 16 | 0.25–16 |
| Tigecycline* | 93.7 | 6.3 | 1 | 2 | 0.25–16 |
| Meropenem | 96.4 | 3.6 | 0.06 | 0.12 | 0.06–32 |
| Amikacin | 93.7 | 6.3 | 2 | 8 | 0.25–128 |

1. **Europe Region**

| **Antimicrobial agent** | **Susceptibility** | | **MIC (mg/L)** | | |
| --- | --- | --- | --- | --- | --- |
|  | **%S** | **%R** | **MIC_50_** | **MIC_90_** | **MIC range** |
| **All Enterobacterales (N = 6816)** | | | | | |
| Aztreonam/avibactam | 99.9 | 0.1 | 0.06 | 0.25 | 0.015–64 |
| Aztreonam | 76.5 | 23.5 | 0.12 | 64 | 0.03–128 |
| Cefepime | 82.5 | 17.5 | 0.12 | 32 | 0.12–64 |
| Colistin | 82.0 | 17.9 | 0.25 | 16 | 0.12–16 |
| Tigecycline* | 97.3 | 2.7 | 0.5 | 1 | 0.03–16 |
| Meropenem | 94.8 | 5.2 | 0.06 | 0.12 | 0.06–32 |
| Amikacin | 95 | 5 | 2 | 4 | 0.25–128 |
| ***Enterobacter cloacae*** | | | | | |
| Aztreonam/avibactam | 100 | 0 | 0.06 | 0.5 | 0.015–2 |
| Aztreonam | 68.5 | 31.5 | 0.12 | 64 | 0.03–128 |
| Cefepime | 87.8 | 12.2 | 0.12 | 16 | 0.12–64 |
| Colistin | 90.3 | 9.6 | 0.25 | 1 | 0.12–16 |
| Tigecycline* | 97.0 | 3.0 | 0.5 | 1 | 0.12–4 |
| Meropenem | 99.0 | 1.0 | 0.06 | 0.12 | 0.06–16 |
| Amikacin | 99.0 | 1.0 | 1 | 2 | 0.5–16 |
| ***Escherichia coli*** | | | | | |
| Aztreonam/avibactam | 99.8 | 0.2 | 0.03 | 0.06 | 0.015–16 |
| Aztreonam | 83.4 | 16.6 | 0.12 | 32 | 0.03–128 |
| Cefepime | 86.4 | 13.6 | 0.12 | 16 | 0.12–64 |
| Colistin | 99.6 | 0.4 | 0.12 | 0.25 | 0.12–16 |
| Tigecycline* | 99.8 | 0.2 | 0.12 | 0.25 | 0.03–4 |
| Meropenem | 99.9 | 0.1 | 0.06 | 0.06 | 0.06–32 |
| Amikacin | 98.2 | 1.7 | 2 | 4 | 0.25–128 |
| ***Klebsiella pneumoniae*** | | | | | |
| Aztreonam/avibactam | 99.9 | 0.1 | 0.06 | 0.25 | 0.015–32 |
| Aztreonam | 58.8 | 41.2 | 0.25 | 128 | 0.03–128 |
| Cefepime | 59.8 | 40.2 | 0.12 | 64 | 0.12–64 |
| Colistin | 91.3 | 8.7 | 0.25 | 1 | 0.12–16 |
| Tigecycline* | 96.4 | 3.6 | 0.5 | 2 | 0.12–16 |
| Meropenem | 83.5 | 16.5 | 0.06 | 32 | 0.06–32 |
| Amikacin | 86.8 | 13.1 | 1 | 32 | 0.25–128 |
| ***Proteus mirabilis*** |  |  |  |  |  |
| Aztreonam/avibactam | 100 | 0 | 0.015 | 0.015 | 0.015–4 |
| Aztreonam | 97.7 | 2.3 | 0.03 | 2 | 0.03–32 |
| Cefepime | 92.7 | 7.3 | 0.12 | 4 | 0.12–64 |
| Colistin | 0 | 100 | 16 | 16 | 8–16 |
| Tigecycline* | 77.0 | 23.0 | 2 | 4 | 0.25–8 |
| Meropenem | 99.4 | 0.6 | 0.06 | 0.12 | 0.06–32 |
| Amikacin | 91.6 | 8.4 | 4 | 8 | 1–128 |
| ***Serratia marcescens*** |  |  |  |  |  |
| Aztreonam/avibactam | 100 | 0 | 0.06 | 0.12 | 0.03–2 |
| Aztreonam | 97.0 | 3.0 | 0.12 | 0.5 | 0.03–128 |
| Cefepime | 97.2 | 2.8 | 0.12 | 0.25 | 0.12–64 |
| Colistin | 4.8 | 95.2 | 16 | 16 | 0.25–16 |
| Tigecycline* | 99.5 | 0.5 | 1 | 2 | 0.25–4 |
| Meropenem | 99.7 | 0.3 | 0.06 | 0.06 | 0.06–32 |
| Amikacin | 99.2 | 0.8 | 2 | 4 | 0.5–16 |

1. **Latin America Region**

| **Antimicrobial Agent** | **EUCAST** | | **MIC (mg/L)** | | |
| --- | --- | --- | --- | --- | --- |
|  | **%S** | **%R** | **MIC_50_** | **MIC_90_** | **MIC range** |
| **All Enterobacterales (N = 1925)** | | | | | |
| Aztreonam/avibactam | 99.8 | 0.2 | 0.06 | 0.25 | 0.015–128 |
| Aztreonam | 62.7 | 37.3 | 0.12 | 128 | 0.03–128 |
| Cefepime | 67.8 | 32.2 | 0.12 | 64 | 0.12–64 |
| Colistin | 80.9 | 19.1 | 0.25 | 16 | 0.12–16 |
| Tigecycline* | 97.5 | 2.5 | 0.5 | 1 | 0.03–8 |
| Meropenem | 92.2 | 7.8 | 0.06 | 4 | 0.06–32 |
| Amikacin | 91.0 | 9.0 | 2 | 8 | 0.25–128 |
| ***Enterobacter cloacae*** | | | | | |
| Aztreonam/avibactam | 100 | 0 | 0.06 | 0.5 | 0.015–2 |
| Aztreonam | 65.7 | 34.3 | 0.25 | 128 | 0.03–128 |
| Cefepime | 80.6 | 19.4 | 0.12 | 32 | 0.12–64 |
| Colistin | 82.1 | 17.9 | 0.25 | 16 | 0.12–16 |
| Tigecycline* | 98.5 | 1.5 | 0.5 | 1 | 0.25–4 |
| Meropenem | 94.0 | 6.0 | 0.06 | 0.25 | 0.06–32 |
| Amikacin | 95.5 | 4.5 | 1 | 4 | 1–128 |
| ***Escherichia coli*** | | | | | |
| Aztreonam/avibactam | 99.6 | 0.4 | 0.03 | 0.12 | 0.015–128 |
| Aztreonam | 59.8 | 40.2 | 0.12 | 64 | 0.03–128 |
| Cefepime | 66.0 | 34.0 | 0.12 | 64 | 0.12–64 |
| Colistin | 99.0 | 1.0 | 0.25 | 0.25 | 0.12–16 |
| Tigecycline* | 98.1 | 1.9 | 0.25 | 0.5 | 0.03–4 |
| Meropenem | 97.3 | 2.7 | 0.06 | 0.06 | 0.06–32 |
| Amikacin | 93.4 | 6.6 | 2 | 8 | 0.25–128 |
| ***Klebsiella pneumoniae*** | | | | | |
| Aztreonam/avibactam | 100 | 0 | 0.06 | 0.25 | 0.015–4 |
| Aztreonam | 45.9 | 54.1 | 16 | 128 | 0.03–128 |
| Cefepime | 48.5 | 51.5 | 8 | 64 | 0.12–64 |
| Colistin | 91.9 | 8.1 | 0.25 | 1 | 0.12–16 |
| Tigecycline* | 97.8 | 2.2 | 0.5 | 2 | 0.12–4 |
| Meropenem | 84.9 | 15.1 | 0.06 | 32 | 0.06–32 |
| Amikacin | 85.8 | 14.2 | 2 | 32 | 0.25–128 |
| ***Proteus mirabilis*** |  |  |  |  |  |
| Aztreonam/avibactam | 98.0 | 2 | 0.015 | 0.015 | 0.015–128 |
| Aztreonam | 98.0 | 2 | 0.03 | 0.25 | 0.03–128 |
| Cefepime | 94.0 | 6 | 0.12 | 2 | 0.12–64 |
| Colistin | 0 | 100 | 16 | 16 | 16 |
| Tigecycline* | 80.0 | 20.0 | 2 | 4 | 0.5–4 |
| Meropenem | 100.0 | 0 | 0.06 | 0.12 | 0.06–4 |
| Amikacin | 94 | 6 | 4 | 8 | 2–128 |
| ***Serratia marcescens*** |  |  |  |  |  |
| Aztreonam/avibactam | 100 | 0 | 0.06 | 0.25 | 0.015–4 |
| Aztreonam | 85.0 | 15.0 | 0.12 | 128 | 0.03–128 |
| Cefepime | 88.2 | 11.8 | 0.12 | 8 | 0.12–64 |
| Colistin | 5.5 | 94.5 | 16 | 16 | 0.25–16 |
| Tigecycline* | 95.3 | 4.7 | 1 | 2 | 0.25–8 |
| Meropenem | 92.1 | 7.9 | 0.06 | 4 | 0.06–32 |
| Amikacin | 87.4 | 12.6 | 2 | 16 | 1–128 |
| cIAI, complicated Intra-Abdominal Infections; EUCAST, European Committee on Antimicrobial Susceptibility Testing; HAP, Hospital-Acquired Pneumonia; MIC, Minimum Inhibitory Concentration; MIC_50_, minimum concentration required to inhibit 50% of the organisms; MIC_90_, minimum concentration required to inhibit 90% of the organisms; S, Susceptibility; R, Resistance; VAP, Ventilator-Acquired Pneumonia  Captures data from Africa and Middle East, Asia-Pacific, Europe and LATAM.  EUCAST 2025 (v15.0) approved breakpoints have been used in this analysis.  %S data includes percentage isolates susceptible at increased exposure.  *Data for tigecycline reported here were calculated based on FDA approved breakpoints. | | | | | |

**Supplementary Table 7. Phenotype-based antimicrobial activities of aztreonam/avibactam and comparators from HAP, VAP, and cIAI across regions (2021-2022).**

| **Antimicrobial agent** | **Susceptibility (EUCAST)** | | **MIC (mg/L)** | | |
| --- | --- | --- | --- | --- | --- |
|  | **%S** | **%R** | **MIC_50_** | **MIC_90_** | **MIC range** |
| **MDR (n = 6217)** | | | | | |
| **AfME (n = 567)** | | | | | |
| Amikacin | 84.1 | 15.9 | 2 | 16 | 0.25–128 |
| Aztreonam | 27.7 | 72.3 | 32 | 128 | 0.03–128 |
| Aztreonam/avibactam | 99.3 | 0.7 | 0.06 | 0.25 | 0.015–128 |
| Cefepime | 38.6 | 61.4 | 8 | 64 | 0.12–64 |
| Colistin | 86.1 | 13.9 | 0.25 | 16 | 0.12–16 |
| Meropenem | 86.8 | 13.2 | 0.06 | 32 | 0.06–32 |
| Tigecycline* | 95.2 | 4.8 | 0.5 | 2 | 0.06–8 |
| **APAC (n = 2294)** | | | | | |
| Amikacin | 79.0 | 21.0 | 2 | 128 | 0.25–128 |
| Aztreonam | 30.3 | 67.7 | 32 | 128 | 0.03–128 |
| Aztreonam/avibactam | 97.8 | 2.2 | 0.12 | 1 | 0.015–128 |
| Cefepime | 42.0 | 58.0 | 16 | 64 | 0.12–64 |
| Colistin | 84.9 | 15.1 | 0.25 | 16 | 0.12–16 |
| Meropenem | 78.0 | 22.0 | 0.06 | 32 | 0.06–32 |
| Tigecycline* | 90.5 | 9.5 | 0.5 | 2 | 0.06–16 |
| **Europe (n = 2404)** | | | | | |
| Amikacin | 86.1 | 13.9 | 2 | 32 | 0.25–128 |
| Aztreonam | 33.6 | 66.4 | 32 | 128 | 0.03–128 |
| Aztreonam/avibactam | 99.7 | 0.3 | 0.12 | 0.5 | 0.015–64 |
| Cefepime | 50.6 | 49.4 | 4 | 64 | 0.12–64 |
| Colistin | 81.1 | 18.9 | 0.25 | 16 | 0.12–16 |
| Meropenem | 85.2 | 14.8 | 0.06 | 32 | 0.06–32 |
| Tigecycline* | 95.6 | 4.4 | 0.5 | 2 | 0.06–16 |
| **LATAM (n = 952)** | | | | | |
| Amikacin | 81.9 | 18.1 | 4 | 32 | 0.25–128 |
| Aztreonam | 24.9 | 75.1 | 32 | 128 | 0.03–128 |
| Aztreonam/avibactam | 99.7 | 0.3 | 0.06 | 0.5 | 0.015–128 |
| Cefepime | 35.1 | 64.9 | 16 | 64 | 0.12–64 |
| Colistin | 81.4 | 18.6 | 0.25 | 16 | 0.12–16 |
| Meropenem | 84.1 | 15.9 | 0.06 | 32 | 0.06–32 |
| Tigecycline* | 97.1 | 2.9 | 0.5 | 2 | 0.03–8 |
| **ESBL (n = 1688)** | | | | | |
| **AfME (n = 197)** | | | | | |
| Amikacin | 73.6 | 26.4 | 4 | 128 | 0.25–128 |
| Aztreonam | 2.5 | 97.5 | 64 | 128 | 2–128 |
| Aztreonam/avibactam | 99.5 | 0.5 | 0.06 | 0.25 | 0.015–128 |
| Cefepime | 11.7 | 88.3 | 64 | 64 | 0.5–64 |
| Colistin | 90.9 | 9.1 | 0.25 | 2 | 0.12–16 |
| Meropenem | 68.0 | 32.0 | 0.06 | 32 | 0.06–32 |
| Tigecycline* | 100 | 0 | 0.5 | 2 | 0.12–2 |
| **APAC (n = 488)** | | | | | |
| Amikacin | 50.6 | 49.4 | 8 | 128 | 0.5–128 |
| Aztreonam | 2.7 | 97.3 | 128 | 128 | 0.5-128 |
| Aztreonam/avibactam | 97.5 | 2.5 | 0.25 | 0.5 | 0.015–128 |
| Cefepime | 6.3 | 93.7 | 64 | 64 | 0.12–64 |
| Colistin | 93.0 | 7.0 | 0.25 | 0.5 | 0.12–64 |
| Meropenem | 44.3 | 55.7 | 32 | 32 | 0.06–32 |
| Tigecycline* | 95.7 | 4.3 | 1 | 2 | 0.06–8 |
| **Europe (n = 667)** | | | | | |
| Amikacin | 71.4 | 28.6 | 4 | 128 | 0.5–128 |
| Aztreonam | 2.1 | 97.9 | 128 | 128 | 0.12–128 |
| Aztreonam/avibactam | 99.7 | 0.3 | 0.12 | 0.5 | 0.015–16 |
| Cefepime | 4.7 | 95.3 | 64 | 64 | 0.12–64 |
| Colistin | 80.7 | 19.3 | 0.25 | 16 | 0.12–16 |
| Meropenem | 67.0 | 33.0 | 0.06 | 32 | 0.06–32 |
| Tigecycline* | 96.9 | 3.1 | 0.5 | 2 | 0.06–16 |
| **LATAM (n = 336)** | | | | | |
| Amikacin | 75 | 25 | 4 | 128 | 0.5–128 |
| Aztreonam | 2.1 | 97.9 | 128 | 128 | 2–128 |
| Aztreonam/avibactam | 99.7 | 0.3 | 0.12 | 0.5 | 0.015–128 |
| Cefepime | 7.4 | 92.6 | 64 | 64 | 0.5–64 |
| Colistin | 86.3 | 13.7 | 0.25 | 8 | 0.12–16 |
| Meropenem | 74.4 | 25.6 | 0.06 | 32 | 0.06–32 |
| Tigecycline* | 98.2 | 1.8 | 0.5 | 1 | 0.06–4 |
| **CRE (n = 1088)** | | | | | |
| **AfME (n = 75)** | | | | | |
| Amikacin | 36 | 64 | 16 | 128 | 1–128 |
| Aztreonam | 8 | 92 | 128 | 128 | 0.03–128 |
| Aztreonam/avibactam | 97.3 | 2.7 | 0.25 | 0.5 | 0.015–128 |
| Cefepime | 1.3 | 98.7 | 64 | 64 | 4–64 |
| Colistin | 74.7 | 25.3 | 0.25 | 16 | 0.12–16 |
| Meropenem | 0 | 100 | 32 | 32 | 16–32 |
| Tigecycline* | 98.7 | 1.3 | 1 | 2 | 0.25–8 |
| **APAC (n = 505)** | | | | | |
| Amikacin | 30.1 | 69.9 | 128 | 128 | 0.5–128 |
| Aztreonam | 6.7 | 93.3 | 128 | 128 | 0.12–128 |
| Aztreonam/avibactam | 93.5 | 6.5 | 0.5 | 2 | 0.015–128 |
| Cefepime | 0.8 | 99.2 | 64 | 64 | 1–64 |
| Colistin | 91.9 | 8.1 | 0.25 | 1 | 0.12–16 |
| Meropenem | 0 | 100 | 32 | 32 | 16–32 |
| Tigecycline* | 85.5 | 14.5 | 1 | 4 | 0.12–16 |
| **Europe (n = 357)** | | | | | |
| Amikacin | 33.0 | 67.0 | 32 | 128 | 0.5–128 |
| Aztreonam | 5.9 | 94.1 | 128 | 128 | 0.06–128 |
| Aztreonam/avibactam | 99.2 | 0.8 | 0.25 | 0.5 | 0.015–64 |
| Cefepime | 1.4 | 98.6 | 64 | 64 | 1–64 |
| Colistin | 60.5 | 39.5 | 0.5 | 16 | 0.12–16 |
| Meropenem | 0 | 100 | 32 | 32 | 16–32 |
| Tigecycline* | 93 | 7 | 1 | 2 | 0.25–16 |
| **LATAM (n = 151)** | | | | | |
| Amikacin | 43.7 | 56.3 | 16 | 128 | 0.5–128 |
| Aztreonam | 9.9 | 90.1 | 128 | 128 | 0.03–128 |
| Aztreonam/avibactam | 99.3 | 0.7 | 0.25 | 0.5 | 0.015–128 |
| Cefepime | 0.7 | 99.3 | 64 | 64 | 4–64 |
| Colistin | 59.6 | 40.4 | 0.5 | 16 | 0.12–16 |
| Meropenem | 0 | 100 | 32 | 32 | 16–32 |
| Tigecycline* | 94.7 | 5.3 | 1 | 2 | 0.12–8 |
| Enterobacterales included the following genera: *Citrobacter, Enterobacter, Escherichia, Klebsiella, Proteus, Providencia, Morganella* and *Serratia*  AfME, Africa and Middle East; APAC, Asia-Pacific; cIAI, complicated Intra-Abdominal Infections; EUCAST, European Committee on Antimicrobial Susceptibility Testing; HAP, Hospital-Acquired Pneumonia; MIC, Minimum Inhibitory Concentration; MIC_50_, minimum concentration required to inhibit 50% of the organisms; MIC_90_, minimum concentration required to inhibit 90% of the organisms; S, Susceptibility; R, Resistance; VAP, Ventilator-Acquired Pneumonia  *Data for tigecycline reported here were calculated based on FDA approved breakpoints. | | | | | |

**Supplementary Table 8: Phenotype-based antimicrobial activities of aztreonam/avibactam and comparators for *E. coli* and *K. pneumoniae* isolates from HAP, VAP and cIAI (2021-2022).**

| **Phenotype** | **Antimicrobials** | **%S (EUCAST)** | **% R (EUCAST)** | **MIC (mg/L)** | | |
| --- | --- | --- | --- | --- | --- | --- |
|  |  |  |  | **MIC_50_** | **MIC_90_** | **MIC range** |
| ***Escherichia coli*** | | | | | | |
| **MDR (n = 1729)** | Amikacin | 92.7 | 7.3 | 4 | 8 | 0.25–128 |
|  | Aztreonam | 39.8 | 60.2 | 16 | 128 | 0.03–128 |
|  | Aztreonam/avibactam | 97.5 | 2.5 | 0.06 | 0.25 | 0.015–128 |
|  | Cefepime | 50.8 | 49.1 | 4 | 64 | 0.12–64 |
|  | Colistin | 99.0 | 1.0 | 0.25 | 0.25 | 0.12–16 |
|  | Meropenem | 95.7 | 4.3 | 0.06 | 0.12 | 0.06–32 |
|  | Tigecycline* | 99.4 | 0.6 | 0.25 | 0.5 | 0.03–8 |
| **ESBL (n = 401)** | Amikacin | 88.5 | 11.5 | 4 | 16 | 1–128 |
|  | Aztreonam | 5.0 | 95.0 | 32 | 128 | 0.12–128 |
|  | Aztreonam/avibactam | 97.5 | 2.5 | 0.06 | 0.5 | 0.015–16 |
|  | Cefepime | 16.4 | 83.5 | 32 | 64 | 0.12–64 |
|  | Colistin | 99.0 | 1 | 0.12 | 0.25 | 0.12–4 |
|  | Meropenem | 93.0 | 7.0 | 0.06 | 2 | 0.06–32 |
|  | Tigecycline* | 99.3 | 0.7 | 0.25 | 0.5 | 0.06–4 |
| **CRE (n = 75)** | Amikacin | 68 | 32 | 4 | 128 | 1–128 |
|  | Aztreonam | 6.7 | 93.3 | 64 | 128 | 0.12–128 |
|  | Aztreonam/avibactam | 66.7 | 33.3 | 4 | 8 | 0.015–128 |
|  | Cefepime | 1.3 | 98.7 | 64 | 64 | 1–64 |
|  | Colistin | 98.7 | 1.3 | 0.12 | 0.25 | 0.12–16 |
|  | Meropenem | 0 | 100 | 32 | 32 | 16–32 |
|  | Tigecycline* | 100 | 0 | 0.25 | 0.5 | 0.12–2 |
| ***Klebsiella pneumoniae*** | | | | | | |
| **MDR (n = 2545)** | Amikacin | 69.5 | 30.5 | 4 | 128 | 0.25–128 |
|  | Aztreonam | 13.5 | 86.4 | 128 | 128 | 0.03–128 |
|  | Aztreonam/avibactam | 99.6 | 0.3 | 0.12 | 0.5 | 0.015–128 |
|  | Cefepime | 16.7 | 83.3 | 64 | 64 | 0.12–64 |
|  | Colistin | 89.3 | 10.7 | 0.25 | 4 | 0.12–16 |
|  | Meropenem | 64.9 | 35.1 | 0.12 | 32 | 0.06–32 |
|  | Tigecycline* | 92.9 | 7.1 | 1 | 2 | 0.12–16 |
| **ESBL (n = 1251)** | Amikacin | 59.1 | 40.9 | 4 | 128 | 0.25–128 |
|  | Aztreonam | 0.9 | 99.0 | 128 | 128 | 0.25–128 |
|  | Aztreonam/avibactam | 99.7 | 0.2 | 0.12 | 0.5 | 0.015–128 |
|  | Cefepime | 3.9 | 97.0 | 64 | 64 | 0.12–64 |
|  | Colistin | 83.4 | 16.5 | 0.25 | 8 | 0.12–16 |
|  | Meropenem | 51.3 | 48.7 | 8 | 32 | 0.06–32 |
|  | Tigecycline* | 96.9 | 3.1 | 1 | 2 | 0.12–16 |
| **CRE (n = 893)** | Amikacin | 28 | 72 | 128 | 128 | 0.5–128 |
|  | Aztreonam | 3.9 | 96.1 | 128 | 128 | 0.03–128 |
|  | Aztreonam/avibactam | 99.2 | 0.8 | 0.25 | 1 | 0.015–128 |
|  | Cefepime | 1.0 | 99.0 | 64 | 64 | 1–64 |
|  | Colistin | 76.4 | 23.6 | 0.25 | 16 | 0.12–16 |
|  | Meropenem | 0 | 100 | 32 | 32 | 16–32 |
|  | Tigecycline* | 90.4 | 9.6 | 1 | 2 | 0.12–16 |
| cIAI, complicated Intra-Abdominal Infections; CRE, Carbapenem-Resistant Enterobacterales; ESBL, Extended-Spectrum β-lactamase; EUCAST, European Committee on Antimicrobial Susceptibility Testing; HAP, Hospital-Acquired Pneumonia; LATAM, Latin America; MDR, Multidrug-Resistant; MIC, Minimum Inhibitory Concentration; MIC_50_, minimum concentration required to inhibit 50% of the organisms; MIC_90_, minimum concentration required to inhibit 90% of the organisms; S, Susceptibility; R, Resistance; VAP, Ventilator-Acquired Pneumonia  Enterobacterales included for MDR and CRE: *Citrobacter,* *Enterobacter,* *Escherichia, Klebsiella, Morganella, Proteus, Providencia, and Serratia.*  Enterobacterales that qualified for β-lactamase screening: *Escherichia coli, Klebsiella oxytoca, Klebsiella pneumoniae, Klebsiella variicola,* and *Proteus mirabilis.*  EUCAST 2025 (v15.0) approved breakpoints have been used in this analysis.  %S data includes percentage isolates susceptible at increased exposure.  *Data for tigecycline reported here were calculated based on FDA approved breakpoints. | | | | | | |

**Supplementary Table 9: Genotype-based antimicrobial activities of aztreonam/avibactam and cefiderocol against MBL-positive Enterobacterales from HAP, VAP and cIAI (2021-2022).**

| **Antimicrobial agent** | **Genotype** | **No. of isolates inhibited at respective MIC (mg/L): n (Cumulative percentage of isolates at each MIC)** | | | | | | | | | | | | | **Total** |
| --- | --- | --- | --- | --- | --- | --- | --- | --- | --- | --- | --- | --- | --- | --- | --- |
|  |  | **0.015** | **0.03** | **0.06** | **0.12** | **0.25** | **0.5** | **1** | **2** | **4** | **8** | **16** | **≥32** | **>64** |  |
| **MBL-Positive Enterobacterales (N =577)** | | | | | | | | | | | | | | | |
| **Cefiderocol** | NDM |  |  | 1  (0.2) | 4  (1) | 2 (1.4) | 7 (2.8) | 50 (12.6) | 177 (47.4 | 190 (84.9) | 39 (92.6) | 15 (95.5) | 22 (100) |  | 508 |
|  | VIM |  |  |  |  | 4 (8.3) | 7 (22.9) | 13 (50) | 11 (72.9) | 9 (91.7) | 2 (95.8) | 1 (97.9) | 1  (100) |  | 48 |
|  | IMP |  |  |  | 2  (8) |  | 5  (28) | 5  (48) | 5  (68) | 7  (96) | 1 (100) |  |  |  | 25 |
|  | OXA-48-like |  |  |  |  |  | 1 (0.7) | 23 (16.5) | 65 (61.4) | 47 (93.8) | 5 (97.2) | 2 (98.6) | 2 (100) |  | 145 |
|  | KPC |  |  |  |  |  | 2 (6.7) | 6 (26.7) | 4 (40.0) | 8 (66.7) | 5 (83.3) | 3 (93.3) | 2 (100) |  | 30 |
|  | ESBL |  |  |  |  | 1 (0.3) | 4 (1.4) | 37 (11.9) | 135 (50.1) | 129 (86.7) | 28 (94.6) | 9 (97.2) | 9 (100) |  | 353 |
|  | pAmpC |  |  |  | 1 (1.4) | 2 (4.2) | 2 (6.9) | 6 (15.3) | 16 (37.5) | 29 (77.8) | 3 (81.9) | 3 (86.1) | 10 (100) |  | 72 |
| **Aztreonam/avibactam** | NDM | 8  (1.6) | 27 (6.9) | 42 (15.1) | 69 (28.7) | 147 (57.7) | 120 (81.3) | 35 (88.2) | 12 (90.5) | 20 (94.5) | 15 (97.4) | 7 (98.8) | 5 (99.8) | 1 (100) | 508 |
|  | VIM | 1  (2.1) | 2 (6.2) | 2 (10.4) | 5 (20.8) | 9 (39.6) | 14 (68.7) | 9 (87.5) | 5 (97.9) | 1 (100) |  |  |  |  | 48 |
|  | IMP |  | 3 (12) | 3  (24) | 2  (32) | 7  (60) | 4  (76) | 2  (84) | 2  (92) | 2 (100) |  |  |  |  | 25 |
|  | OXA-48-like |  | 1 (0.7) | 2 (2.0) | 5 (5.5) | 51 (40.7) | 74 (91.7) | 7 (96.5) | 1 (97.2) | 2 (98.6) | 1 (99.3) | 1 (100) |  |  | 145 |
|  | KPC |  |  |  | 1 (3.3) | 7 (26.7) | 10 (60) | 12 (100) |  |  |  |  |  |  | 30 |
|  | ESBL | 1  (0.3) | 10 (3.1) | 18 (8.2) | 41 (19.8) | 119 (53.5) | 107 (83.9) | 30 (92.4) | 7 (94.3) | 16 (98.9) | 3 (99.7) |  | 1 (100) |  | 353 |
|  | pAmpC | 2  (2.8) | 2 (5.6) | 2 (8.3) | 4 (13.9) | 4 (19.4) | 11 (34.7) | 8 (45.8) | 8 (56.9) | 7 (66.7) | 12 (83.3) | 7 (93) | 5 (100) |  | 72 |
| cIAI, complicated Intra-Abdominal Infections; ESBL, Extended-Spectrum β-lactamase; HAP, Hospital-Acquired Pneumonia; IMP, Imipenemase Metallo-β-lactamase; KPC, Klebsiella Pneumoniae Carbapenemase; NDM, New Delhi Metallo-β-lactamase; OXA-48-like, Oxacillinase-48-like β-lactamase; pAmpC, plasmid-mediated ambler class C β-lactamase VAP, Ventilator-Acquired Pneumonia; VIM, Verona Integron-encoded Metallo-β-lactamase. | | | | | | | | | | | | | | | |

**Supplementary Table 10: Genotype-based antimicrobial activities of aztreonam/avibactam and comparators for *E. coli* and *K. pneumoniae* isolates from HAP, VAP and cIAI (2021-2022).**

| \| **Genotypes** \| **n**^†^ \| **Antimicrobial agent** \| \| \| \| \| \| \| \| \| \| \| \| \| --- \| --- \| --- \| --- \| --- \| --- \| --- \| --- \| --- \| --- \| --- \| --- \| --- \| --- \| \| **MIC_90_ (mg/L)/% susceptible (%S), EUCAST** \| \| \| \| \| \| \| \| \| \| \| \| \| **Aztreonam/avibactam** \| \| **Aztreonam** \| \| **Colistin** \| \| **Cefiderocol** \| \| **Cefepime** \| \| **Tigecycline** \| \| \| **%S** \| **MIC_90_** \| **%S** \| **MIC_90_** \| **%S** \| **MIC_90_** \| **%S** \| **MIC_90_** \| **%S** \| **MIC_90_** \| **%S** \| **MIC_90_** \| | | | | | | | | | | | | | |
| --- | --- | --- | --- | --- | --- | --- | --- | --- | --- | --- | --- | --- | --- | --- | --- | --- | --- | --- | --- | --- | --- | --- | --- | --- | --- | --- | --- | --- | --- | --- | --- | --- | --- | --- | --- | --- | --- | --- | --- | --- | --- | --- | --- | --- | --- | --- | --- | --- | --- | --- | --- | --- | --- | --- | --- | --- | --- | --- | --- | --- | --- | --- | --- |
| ***Escherichia coli*** | | | | | | | | | | | | | |
| NDM | 80 | 65 | 16 | 10.0 | >64 | 100 | 0.5 | 33.7 | >32 | 0.0 | >32 | 100.0 | 0.5 |
| IMP | 1 | 100 | 0.06 | 0.0 | >64 | 100 | 0.25 | 100.0 | 0.5 | 0.0 | >32 | 100.0 | 0.12 |
| Co-carriage | | | | | | | | | | | | | |
| OXA-48-like | 4 | 50 | 0.03–16 | 0.0 | >64 | 100 | <0.12–0.25 | 25 | 2->32 | 0.0 | >32 | 100.0 | 0.12–0.5 |
| ESBL | 41 | 90.2 | 4 | 0.0 | >64 | 100 | 0.5 | 39.0 | 16 | 0.0 | >32 | 100.0 | 0.5 |
| pAmpC | 37 | 35.1 | 32 | 0.0 | >64 | 100 | 0.5 | 18.9 | >32 | 0.0 | >32 | 100.0 | 0.5 |
| ***Klebsiella pneumoniae*** | | | | | | | | | | | | | |
| NDM | 324 | 100 | 0.5 | 5.6 | >64 | 72.2 | >8 | 51.5 | 8 | 0.0 | >32 | 99.0 | 2 |
| VIM | 29 | 100 | 1 | 10.3 | >64 | 62.1 | >8 | 79.3 | 4 | 0.0 | >32 | 72.0 | 4 |
| IMP | 3 | 100 | 0.25–1 | 33.3 | 1–>64 | 100 | 0.25 | 100 | 0.5–2 | 0.0 | >32 | 100.0 | 0.5–1 |
| Co-carriage | | | | | | | | | | | | | |
| OXA-48-like | 133 | 100 | 0.5 | 6.8 | >64 | 74.4 | >8 | 64.7 | 4 | 0.0 | >32 | 100.0 | 2 |
| KPC | 28 | 100 | 1 | 0.0 | >64 | 60.7 | >8 | 39.3 | 16 | 0.0 | >32 | 89.3 | 4 |
| ESBL | 306 | 100 | 0.5 | 0.6 | >64 | 71.2 | >8 | 51.6 | 8 | 0.0 | >32 | 98.4 | 2 |
| pAmpC | 30 | 100 | 1 | 3.3 | >64 | 70.0 | >8 | 56.7 | 8 | 0.0 | >32 | 86.7 | 4 |
| cIAI, complicated Intra-Abdominal Infections; ESBL, Extended-Spectrum β-lactamase; HAP, Hospital-Acquired Pneumonia; IMP, Imipenemase Metallo-β-lactamase; KPC, Klebsiella Pneumoniae Carbapenemase; NDM, New Delhi Metallo-β-lactamase; OXA-48-like, Oxacillinase-48-like β-lactamase; pAmpC, plasmid-mediated ambler class C β-lactamase VAP, Ventilator-Acquired Pneumonia; VIM, Verona Integron-encoded Metallo-β-lactamase.  ^†^For entries with fewer than 10 isolates, the MIC range is reported instead of MIC_90_. | | | | | | | | | | | | | |

**Supplementary Table 11: Genotype-based antimicrobial activities of aztreonam/avibactam and comparators against MBL-positive *E. coli* and *K. pneumoniae* isolates from HAP, VAP and cIAI across regions (2021-2022).**

1. **Africa/Middle East Region**

| **Genotypes** | **n**^†^ | **Antimicrobial agent** | | | | | | | | | | | |
| --- | --- | --- | --- | --- | --- | --- | --- | --- | --- | --- | --- | --- | --- |
|  |  | **MIC_90_ (mg/L)/% susceptible (%S), EUCAST** | | | | | | | | | | | |
|  |  | **Aztreonam/avibactam** | | **Aztreonam** | | **Colistin** | | **Cefiderocol** | | **Cefepime** | | **Tigecycline** | |
|  |  | **%S** | **MIC_90_** | **%S** | **MIC_90_** | **%S** | **MIC_90_** | **%S** | **MIC_90_** | **%S** | **MIC_90_** | **%S** | **MIC_90_** |
| **AfME (N = 68)** | | | | | | | | | | | | | |
| NDM | 67 | 100.0 | 0.5 | 9.0 | >64 | 68.7 | >8 | 64.2 | 4 | 0 | >32 | 97.0 | 1 |
| IMP | 1 | 100.0 | 0.25 | 0.0 | 32 | 100.0 | 0.5 | 100.0 | 0.12 | 100.0 | 1 | 100.0 | 0.25 |
| OXA-48-like | 21 | 100.0 | 0.5 | 4.8 | >64 | 66.7 | >8 | 80.9 | 4 | 0 | >32 | 100.0 | 1 |
| ESBL | 54 | 100.0 | 0.5 | 0.0 | >64 | 70.4 | >8 | 63.0 | 4 | 0 | >32 | 98.1 | 1 |
| pAmpC | 3 | 100.0 | 0.03–0.12 | 0.0 | ≥64 | 100.0 | 0.25 | 66.7 | 2–4 | 0 | >32 | 100.0 | 0.25–0.5 |
| ***Escherichia coli*** | | | | | | | | | | | | | |
| NDM | 2 | 100.0 | 0.03–2 | 0.0 | >64 | 100.0 | 0.25 | 50.0 | 2–4 | 0 | >32 | 100.0 | 0.25 |
| OXA-48-like | 1 | 100.0 | 0.03 | 0.0 | >64 | 100.0 | 0.25 | 100.0 | 2 | 0 | >32 | 100.0 | 0.25 |
| ESBL | 1 | 100.0 | 2 | 0.0 | >64 | 100.0 | 0.25 | 0.0 | 4 | 0 | >32 | 100.0 | 0.25 |
| pAmpC | 1 | 100.0 | 0.03 | 0.0 | >64 | 100.0 | 0.25 | 100.0 | 2 | 0 | >32 | 100.0 | 0.25 |
| YRIN | 2 | 100.0 | 0.03–2 | 0.0 | >64 | 100.0 | 0.25 | 50.0 | 2–4 | 0 | >32 | 100.0 | 0.25 |
| YRIN+pAmpC | 1 | 100.0 | 0.03 | 0.0 | >64 | 100.0 | 0.25 | 100.0 | 2 | 0 | >32 | 100.0 | 0.25 |
| ***Klebsiella pneumoniae*** | | | | | | | | | | | | | |
| NDM | 55 | 100.0 | 0.5 | 3.6 | >64 | 69.1 | >8 | 63.6 | 4 | 0 | >32 | 98.2 | 1 |
| OXA-48-like | 20 | 100.0 | 0.5 | 5.0 | >64 | 65.0 | >8 | 80.0 | 4 | 0 | >32 | 100.0 | 1 |
| ESBL | 53 | 100.0 | 0.5 | 0.0 | >64 | 69.8 | >8 | 64.2 | 4 | 0 | >32 | 98.1 | 1 |
| pAmpC | 2 | 100.0 | 0.06–0.12 | 0.0 | ≥64 | 100.0 | 0.25 | 50.0 | 2–4 | 0 | >32 | 100.0 | 0.25–0.5 |

1. **Asia-Pacific Region**

| **Genotypes** | **n**^†^ | **Antimicrobial agent** | | | | | | | | | | | |
| --- | --- | --- | --- | --- | --- | --- | --- | --- | --- | --- | --- | --- | --- |
|  |  | **MIC_90_ (mg/L)/% susceptible (%S), EUCAST** | | | | | | | | | | | |
|  |  | **Aztreonam/avibactam** | | **Aztreonam** | | **Colistin** | | **Cefiderocol** | | **Cefepime** | | **Tigecycline** | |
|  |  | **%S** | **MIC_90_** | **%S** | **MIC_90_** | **%S** | **MIC_90_** | **%S** | **MIC_90_** | **%S** | **MIC_90_** | **%S** | **MIC_90_** |
| **APAC (N = 292)** | | | | | | | | | | | | | |
| NDM | 272 | 89.7 | 8 | 14.3 | >64 | 90.8 | 1 | 49.3 | 16 | 0 | >32 | 97.6 | 2 |
| IMP | 23 | 100.0 | 2 | 26.1 | >64 | 50.0 | 0.25– >8 | 65.2 | 4 | 0 | >32 | 65.2 | >8 |
| OXA-48-like | 98 | 98.0 | 0.5 | 8.2 | >64 | 84.7 | >8 | 62.2 | 4 | 0 | >32 | 99.0 | 2 |
| KPC | 11 | 100.0 | 1 | 0 | >64 | 100.0 | 0.5 | 9.1 | 16 | 0 | >32 | 100.0 | 2 |
| ESBL | 177 | 97.7 | 4 | 56.5 | >64 | 92.7 | 0.5 | 56.5 | 8 | 0 | >32 | 99.4 | 2 |
| pAmpC | 41 | 41.5 | 32 | 2.4 | >64 | 95.1 | 0.5 | 22.0 | >32 | 0 | >32 | 100.0 | 0.5 |
| ***Escherichia coli*** | | | | | | | | | | | | | |
| NDM | 63 | 55.6 | 16 | 3.2 | >64 | 100.0 | 0.5 | 28.6 | >32 | 0 | >32 | 100.0 | 0.5 |
| IMP | 1 | 100.0 | 0.06 | 0 | >64 | 100.0 | 0.25 | 100 | 0.5 | 0 | >32 | 100.0 | 0.12 |
| OXA-48-like | 2 | 0 | 8–16 | 0 | >64 | 100.0 | <0.12–0.25 | 0 | 4–>32 | 0 | >32 | 100.0 | 0.12–0.5 |
| ESBL | 32 | 87.5 | 8 | 0 | >64 | 100.0 | 0.5 | 31.3 | 16 | 0 | >32 | 100.0 | 0.5 |
| pAmpC | 34 | 29.4 | 32 | 0 | >64 | 100.0 | 0.5 | 17.6 | >32 | 0 | >32 | 100.0 | 0.5 |
| YRIK | 21 | 57.1 | 32 | 0 | >64 | 100.0 | 0.5 | 19.0 | 4 | 0 | 0 | 100.0 | 0.5 |
| YRIN | 34 | 44.1 | 16 | 0 | >64 | 100.0 | 0.25 | 17.6 | >32 | >32 | >32 | 100.0 | 0.5 |
| YRIK/YRIN+pAmpC | 33 | 27.3 | 32 | 0 | >64 | 100.0 | 0.25 | 15.2 | >32 | 0 | >32 | 100.0 | 0.5 |
| ***Klebsiella pneumoniae*** | | | | | | | | | | | | | |
| NDM | 157 | 100.0 | 0.5 | 8.3 | >64 | 91.1 | 1 | 60.5 | 4 | 0 | >32 | 98.8 | 2 |
| IMP | 3 | 100.0 | 0.25–1 | 33.3 | 1–>64 | 100.0 | 0.25 | 100.0 | 0.5–2 | 0 | >32 | 100.0 | 0.5–1 |
| OXA-48-like | 91 | 100.0 | 0.5 | 8.8 | >64 | 85.7 | >8 | 65.9 | 4 | 0 | >32 | 100.0 | 2 |
| KPC | 11 | 100.0 | 1 | 0 | >64 | 100.0 | 0.5 | 9.1 | 16 | 0 | >32 | 100.0 | 2 |
| ESBL | 144 | 100.0 | 0.5 | 69.4 | >64 | 91.0 | 1 | 62.5 | 4 | 0 | >32 | 99.3 | 2 |
| pAmpC | 6 | 100.0 | 0.03–4 | 0 | ≥64 | 83.3 | 0.25–4 | 33.3 | 0.5–16 | 0 | >32 | 100.0 | 0.5–1 |

1. **Europe Region**

| **Genotypes** | **n**^†^ | **Antimicrobial agent** | | | | | | | | | | | |
| --- | --- | --- | --- | --- | --- | --- | --- | --- | --- | --- | --- | --- | --- |
|  |  | **MIC_90_ (mg/L)/% susceptible (%S), EUCAST** | | | | | | | | | | | |
|  |  | **Aztreonam/avibactam** | | **Aztreonam** | | **Colistin** | | **Cefiderocol** | | **Cefepime** | | **Tigecycline** | |
|  |  | **%S** | **MIC_90_** | **%S** | **MIC_90_** | **%S** | **MIC_90_** | **%S** | **MIC_90_** | **%S** | **MIC_90_** | **%S** | **MIC_90_** |
| **Europe (N = 145)** | | | | | | | | | | | | | |
| NDM | 99 | 100.0 | 1 | 4.0 | >64 | 39.4 | >8 | 22.2 | 8 | 0.0 | >32 | 98.0 | 2 |
| VIM | 45 | 100.0 | 1 | 15.6 | >64 | 75.5 | >8 | 77.8 | 4 | 0.0 | >32 | 82.2 | 4 |
| IMP | 1 | 100.0 | 0.5 | 0.0 | 64 | 100.0 | 0.25 | 100 | 1 | 100.0 | 1 | 100.0 | 0.25 |
| OXA-48-like | 25 | 100.0 | 1 | 0.0 | >64 | 36.0 | >8 | 44.0 | 8 | 0.0 | >32 | 100.0 | 2 |
| KPC | 16 | 100.0 | 1 | 0.0 | >64 | 43.7 | >8 | 62.5 | 16 | 0.0 | >32 | 81.3 | 4 |
| ESBL | 85 | 100.0 | 1 | 0.0 | >64 | 42.4 | >8 | 22.4 | 8 | 0.0 | >32 | 98.8 | 2 |
| pAmpC | 21 | 100.0 | 2 | 5.0 | >64 | 66.7 | >8 | 66.7 | 4 | 0.0 | >32 | 81.0 | 4 |
| ***Klebsiella pneumoniae*** | | | | | | | | | | | | | |
| NDM | 82 | 100.0 | 1 | 1.2 | >64 | 41.4 | >8 | 23.2 | 8 | 0.0 | >32 | 100.0 | 1 |
| VIM | 28 | 100.0 | 2 | 10.7 | >64 | 64.3 | >8 | 82.1 | 4 | 0.0 | >32 | 71.4 | 4 |
| OXA-48-like | 22 | 100.0 | 0.5 | 0.0 | >64 | 36.4 | >8 | 45.5 | 8 | 0.0 | >32 | 100.0 | 2 |
| KPC | 15 | 100.0 | 1 | 0.0 | >64 | 40.0 | >8 | 60.0 | 16 | 0.0 | >32 | 80.0 | 4 |
| ESBL | 81 | 100.0 | 1 | 0.0 | >64 | 40.7 | >8 | 19.8 | 8 | 0.0 | >32 | 98.8 | 2 |
| pAmpC | 17 | 100.0 | 2 | 6.0 | >64 | 64.7 | >8 | 70.6 | 4 | 0.0 | >32 | 76.5 | 4 |

1. **Latin America Region**

| **Genotypes** | **n**^†^ | **Antimicrobial agent** | | | | | | | | | | | |
| --- | --- | --- | --- | --- | --- | --- | --- | --- | --- | --- | --- | --- | --- |
|  |  | **MIC_90_ (mg/L)/% susceptible (%S), EUCAST** | | | | | | | | | | | |
|  |  | **Aztreonam/avibactam** | | **Aztreonam** | | **Colistin** | | **Cefiderocol** | | **Cefepime** | | **Tigecycline** | |
|  |  | **%S** | **MIC_90_** | **%S** | **MIC_90_** | **%S** | **MIC_90_** | **%S** | **MIC_90_** | **%S** | **MIC_90_** | **%S** | **MIC_90_** |
| **LATAM (N = 72)** | | | | | | | | | | | | | |
| NDM | 70 | 100.0 | 1 | 30.0 | >64 | 68.6 | >8 | 60.0 | 4 | 0 | >32 | 94.3 | 2 |
| VIM | 3 | 100.0 | 0.5–1 | 0 | >64 | 33.3 | 0.5–>8 | 0 | 4–>32 | 0 | >32 | 67.0 | 1–8 |
| OXA-48-like | 1 | 100.0 | 2 | 0 | >64 | 100.0 | 0.25 | 0 | 4 | 0 | >32 | 100.0 | 0.25 |
| KPC | 3 | 100.0 | 0.25–1 | 0 | >64 | 100.0 | >8 | 33.3 | 2– >32 | 0 | >32 | 67.0 | 1–8 |
| ESBL | 37 | 100.0 | 1 | 2.7 | >64 | 70.3 | >8 | 64.9 | 4 | 0 | >32 | 94.6 | 2 |
| pAmpC | 7 | 100.0 | 0.12–2 | 0 | 4– >64 | 71.4 | 0.25– >8 | 28.6 | 2–4 | 0 | ≥32 | 100.0 | 0.25–1 |
| ***Escherichia coli*** | | | | | | | | | | | | | |
| NDM | 15 | 100.0 | 1 | 40.0 | >64 | 100.0 | 0.25 | 53.3 | 4 | 0 | >32 | 100.0 | 0.25 |
| OXA-48-like | 1 | 100.0 | 2 | 0 | >64 | 100.0 | 0.25 | 0 | 4 | 0 | >32 | 100.0 | 0.25 |
| ESBL | 8 | 100.0 | 0.06–2 | 0 | 32–>64 | 100.0 | 0.25 | 75.0 | 1–4 | 0 | >32 | 100.0 | 0.12–0.25 |
| pAmpC | 2 | 100.0 | 0.25–2 | 0 | 16–>64 | 100.0 | 0.25 | 0 | 4 | 0 | >32 | 100.0 | 0.25 |
| YRIN | 4 | 100.0 | 0.5– 2 | 25.0 | 1– >64 | 100.0 | 0.25 | 25.0 | 2–4 | 0 | >32 | 100.0 | 0.25 |
| YRIN+pAmpC | 1 | 100.0 | 2 | 0 | >64 | 100.0 | 0.25 | 0 | 4 | 0 | >32 | 100.0 | 0.25 |
| ***Klebsiella pneumoniae*** | | | | | | | | | | | | | |
| NDM | 30 | 100.0 | 0.5 | 6.7 | >64 | 63.3 | >8 | 60.0 | 4 | 0 | >32 | 93.3 | 2 |
| VIM | 1 | 100.0 | 0.5 | 0 | >64 | 0 | >8 | 0 | 4 | 0 | >32 | 100.0 | 1 |
| KPC | 2 | 100.0 | 0.25–0.5 | 0 | >64 | 100.0 | >8 | 50.0 | 2–4 | 0 | >32 | 100.0 | 1 |
| ESBL | 28 | 100.0 | 0.5 | 3.6 | >64 | 60.7 | >8 | 64.3 | 4 | 0 | >32 | 92.9 | 2 |
| pAmpC | 5 | 100.0 | 0.12–1 | 0 | 4–>64 | 60.0 | 0.25–>8 | 40.0 | 2–4 | 0 | ≥32 | 100.0 | 0.25–1 |
| cIAI, complicated Intra-Abdominal Infections; HAP, Hospital-Acquired Pneumonia; IMP, Imipenemase Metallo-β-lactamas ; KPC, Klebsiella Pneumoniae Carbapenemase; LATAM, Latin America; NDM, New Delhi Metallo-β-lactamase; OXA-48-like, Oxacillinase-48-like β-lactamase; pAmpC, plasmid-mediated ambler class C β-lactamase; VAP, Ventilator-Acquired Pneumonia; VIM, Verona Integron-encoded Metallo-β-lactamase.  ^†^ For entries with fewer than 10 isolates, the MIC range is reported instead of MIC_90_. | | | | | | | | | | | | | |

**Supplementary Table 12: Genotype-based antimicrobial activities of aztreonam/avibactam against MBL-positive *E.coli* isolates from HAP, VAP and cIAI for countries in Asia region** **(2021-2022).**

| **Genotypes** | **Asia Countries (n = 60)** | | | | | | | | |
| --- | --- | --- | --- | --- | --- | --- | --- | --- | --- |
|  | **MIC_90_ (mg/L)/% susceptible (%S), EUCAST** | | | | | | | | |
|  | **China Mainland (n = 9)** | | | **India (n = 49)** | | | **Thailand (n = 2)** | | |
|  | **n**^†^ | **%S** | **MIC_90_** | **n** | **%S** | **MIC_90_** | **n** | **%S** | **MIC_90_** |
| ***Escherichia coli*** | | | | | | | | | |
| NDM | 8 | 87.5 | 0.03–8 | 49 | 46.9 | 32 | 2 | 100 | 0.12–2 |
| IMP | 1 | 100 | 0.06 | - | - | - | - | - | - |
| ESBL | 8 | 87.5 | 0.06–8 | 19 | 89.5 | 8 | 2 | 100 | 0.12–2 |
| pAmpC | - | - | - | 30 | 23.3 | 32 | 2 | 100 | 0.12–2 |
| NDM-5+pAmpC | - | - | - | 24 | 25.0 | 32 | 1 | 100 | 2 |
| cIAI, complicated Intra-Abdominal Infections; HAP, Hospital-Acquired Pneumonia; IMP, Imipenemase Metallo-β-lactamase; KPC, Klebsiella pneumoniae Carbapenemase; LATAM, Latin America; NDM, New Delhi Metallo-β-lactamase; OXA-48-like, Oxacillinase-48-like β-lactamase; pAmpC, plasmid-mediated ambler class C β-lactamase;VAP, Ventilator-Acquired Pneumonia.  ^†^For entries with fewer than 10 isolates, the MIC range is reported instead of MIC_90_. | | | | | | | | | |
